# *Glycyrrhiza glabra* exhibits disease-modifying effects in Alzheimer’s disease through restoration of mitochondrial function, synaptic plasticity, and kinase signaling

**DOI:** 10.3389/fphar.2026.1894678

**Published:** 2026-08-28

**Authors:** S. Amrutha, Manvitha Kadandelu, Murali Krishna Paidi, Shamprasad Varija Raghu, Thottethodi Subrahmanya Keshava Prasad, Prashant Kumar Modi

**Affiliations:** 1 Center for Systems Biology and Molecular Medicine, Yenepoya Research Centre, Yenepoya (Deemed to Be University), Mangalore, India; 2 Division of Neuroscience, Yenepoya Research Centre, Yenepoya (Deemed to Be University), Mangalore, India

**Keywords:** apoptosis, *Glycyrrhiza glabra*, medhya rasayana, mitochondrial dysfunction, neuroprotection

## Abstract

**Background:**

Alzheimer’s disease (AD) is a progressive neurodegenerative disorder characterized by cognitive decline, memory impairment, and neuronal dysfunction. Current therapeutic treatments are limited to symptomatic relief and often cause significant adverse effects, underscoring the need for alternative medicinal approaches with potent neuroprotective efficacy and minimal side effects. *Glycyrrhiza glabra*, rich in major bioactive constituents such as Glycyrrhizic acid, glabridin, liquiritin, rhamnoliquirilin, liquiritigenin, and prenyllicoflavone A, has been historically used in Ayurveda and exhibits therapeutic potential; however, its efficacy in mitigating AD-associated pathological conditions remains poorly understood.

**Methods:**

This study investigates the neuroprotective properties of *G. glabra* root extract in Aβ_42_-treated human neuroblastoma (IMR-32) cells and Aβ_42_-overexpressing *Drosophila melanogaster* transgenic flies. Various behavioral and molecular biology techniques were employed, including immunofluorescence, qRT-PCR, flow cytometry, and western blotting, to investigate the effects of *G. glabra* root extract.

**Results:**

Our results demonstrated that Aβ_42_ treatment induced aberrant alterations in several proteins associated with Alzheimer’s pathology, mitochondrial dysfunction, cell-cycle re-entry, and synaptic activity. Co-treatment with *G. glabra* extract restored these protein levels and mitigated Aβ_42_-induced hyperactivation of key signaling pathways. Furthermore, *G. glabra* attenuated Aβ_42_-induced apoptotic signaling, modulating pro- and anti-apoptotic protein expression profiles in both *in vitro* and *in vivo* models.

**Conclusion:**

Our findings indicate that *G. glabra* treatment restores the expression of key proteins altered in AD pathology, suggesting a potential neuroprotective mechanism mediated through the regulation of the MEK/ERK-1/2, PI3K/AKT/GSK3β, and CDK5/p35/p25 pathways. This study provides a molecular rationale for the historical use of *G. glabra*, demonstrating its ability to rescue altered AD-associated protein markers in both cellular and *Drosophila* models. While further research is necessary to fully elucidate the neuroprotective mechanisms of *G. glabra*, these findings pave the way for downstream validation of the extract as a promising natural therapeutic candidate for Alzheimer’s disease.

## Graphical Abstract

**Figure:**
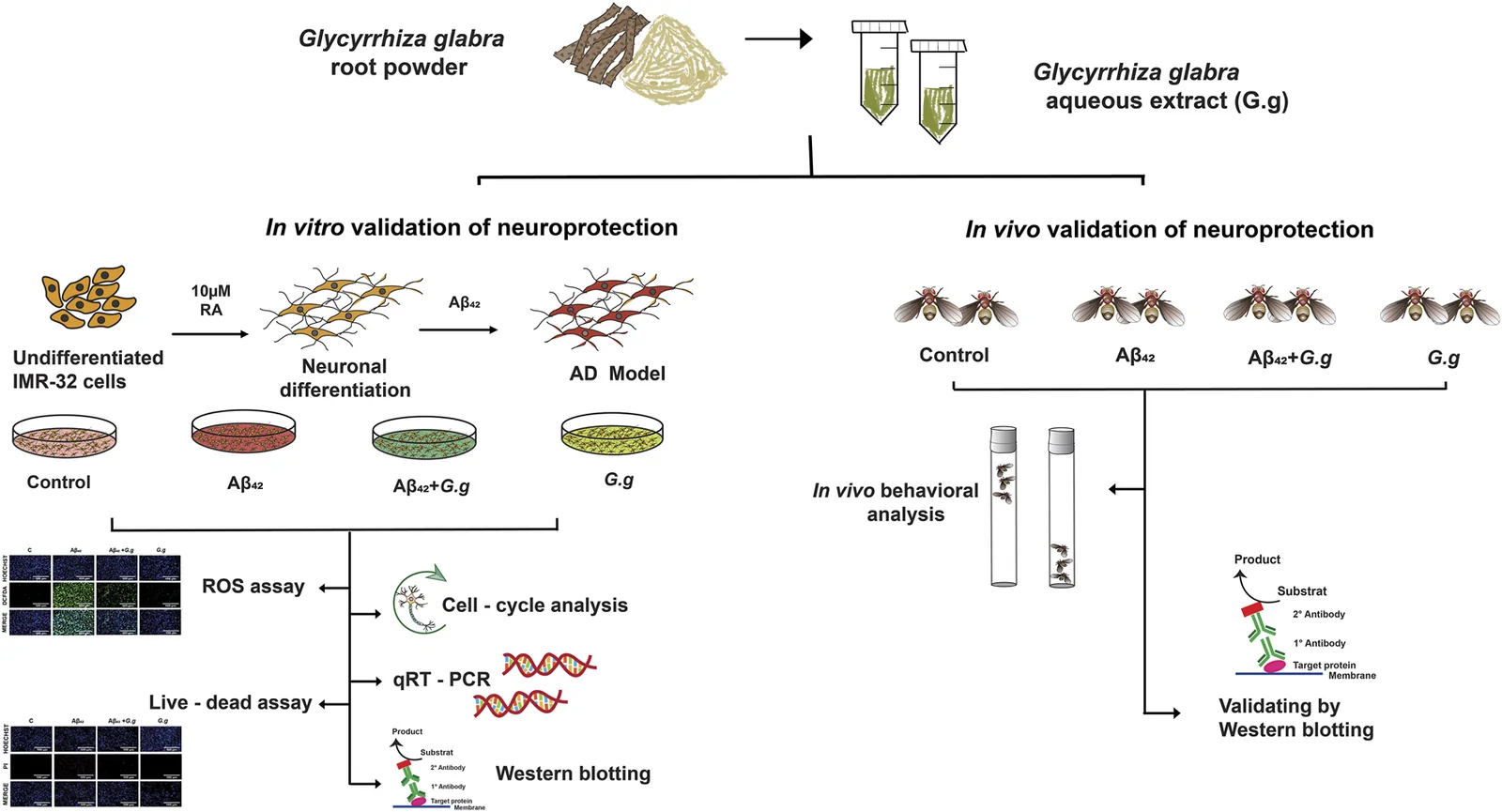

## Introduction

Alzheimer’s disease (AD), a progressive neurodegenerative disorder, is the leading cause of dementia, significantly impacts the elderly population, and is not a normal part of aging. Currently affecting an estimated 50 million people worldwide, this number is expected to increase to 150 million by 2050 (Scheltens et al., 2021; Collaborators, 2022). AD has emerged as a significant global health concern, ranking among the top ten causes of death worldwide, with no specified diagnosis and disease-altering treatments. AD is characterized by memory impairment and cognitive decline, which can ultimately affect behavior, mood, speech, sleep, short-term memory loss, impaired reasoning, poor judgment, language dysfunction, olfactory dysfunction, visuospatial orientation, and motor function, leading to difficulty performing even the simplest tasks (DeTure and Dickson, 2019; Teixeira et al., 2023).

Amyloid beta (Aβ_42_) is a primary causative agent of AD pathology. The accumulation of Aβ_42_ leads to neurofibrillary tangles, neuronal loss, vascular damage, and dementia. An imbalance between Aβ_42_ production and clearance results in the accumulation of various Aβ_42_ isoforms, including protofibrils, fibrils, and plaques. Neurofibrillary tangles, another hallmark of AD, are composed of Tau protein, which normally stabilizes microtubules in neurons. In AD, Tau becomes hyperphosphorylated, loses its affinity for microtubules, and forms neurofibrillary tangles, leading to neuronal dysfunction and death (Arnsten et al., 2021). Several risk factors, such as neurotransmitter dysregulation, inflammation, oxidative stress, mitochondrial dysfunction, and elevated cholesterol, contribute to the development and progression of AD. Altered neurotransmission involves cholinergic dysfunction, increased glutamate release, altered N-methyl-D-aspartate (NMDA) receptor function, and abnormal functioning of gamma-aminobutyric acid (GABA), histamine, and serotonin, all of which contribute to the pathogenesis of AD (Yang et al., 2023).

Currently, available treatments for AD primarily target cholinergic or glutamatergic neurotransmission, offering symptomatic relief rather than a cure. Acetylcholinesterase inhibitors, such as donepezil, rivastigmine, and galantamine, are approved for mild to moderate AD. These drugs work by increasing acetylcholine levels in the brain. However, their clinical utility is limited in patients with chronic renal/liver dysfunction (Hansen et al., 2008). In cases of moderate to severe AD, the NMDA receptor antagonist memantine serves as the primary therapeutic option. Nevertheless, it is frequently associated with adverse effects, including dizziness, body aches, headache, and constipation (Soni et al., 2025). FDA has approved several monoclonal antibodies targeting amyloid beta protein, including aducanumab, lecanemab, and donanemab, for the treatment of Alzheimer’s disease. Although aducanumab was recently discontinued, other FDA-approved drugs, such as lecanemab and donanemab, have shown promising capacity in plaque clearance and slowing cognitive decline in early-AD patients (Gupta and Chaudhuri, 2025). Despite these advancements, there is still an unmet clinical need for discovery of new drugs for treatment of AD. These modern disease-modifying therapies are designed for early clinical stages. Their high cost and potential side effects include amyloid-related imaging abnormalities (ARIA), vasogenic edema (ARIA-E), or microhemorrhage (ARIA-H), headache, confusion, delirium, altered mental status, disorientation, dizziness, vision abnormalities, and nausea, leading to limited accessibility. Furthermore, patients require definitive confirmation of amyloid pathology via costly serial MRI monitoring or expensive positron emission tomography (PET) imaging, which further limits their accessibility to the general population (Tolar et al., 2020; Soderberg et al., 2023; Vitek et al., 2023; Gupta and Chaudhuri, 2025; Soni et al., 2025).

Modern Alzheimer’s disease research has increasingly shifted focus toward targeting mitochondrial dysfunction as a primary therapeutic strategy. Consequently, plant-based interventions that rescue mitochondrial homeostasis have rapidly emerged as a prominent front in drug discovery. Recent studies indicate that natural plant products (NPPs) such as urolithin A and β-asarone can restore mitochondrial health by activating PINK1/Parkin-mediated mitophagy, thereby mitigating Aβ-induced neurotoxicity and improving cognitive function (Yang et al., 2024; Shao et al., 2026). Furthermore, phytochemicals such as gastrodin have been shown to directly rescue mitochondrial energy metabolism and protect hippocampal tissue by restoring vital survival and metabolic markers, including active β-catenin, c-Myc, and MCT2 (Lei et al., 2026).

*Glycyrrhiza glabra L.* is recognized as a pivotal botanical drug in Ayurveda, the traditional Indian medical system. In the Ayurvedic system, it is classified as a Medhya Rasayana, a class of botanical drugs used to treat neurological disorders and enhance cognitive health (http://www.theplantlist.org) (Kulkarni et al., 2012). It is also a central component of Traditional Chinese Medicine (TCM) and is documented in historical Chinese pharmacopeias. Its significance is captured by the traditional belief that “nine out of ten formulae contain licorice,” where it serves as a primary agent for reducing toxicity and harmonizing the effects of other botanical drugs (Wahab et al., 2021). *G. glabra* L., commonly known as licorice or Yashtimadhu, belongs to the Fabaceae family (El-Saber Batiha et al., 2020). The root of *G. glabra* has broad medicinal properties and is used to manage various conditions, ranging from respiratory disorders and gastric ulcers to neurological issues such as epilepsy and paralysis. It is also frequently utilized for jaundice, hyperdipsia, rheumatism, and dermatological or hemorrhagic disorders. Modern pharmacological evaluations have further validated its capacity to act as a potent anti-inflammatory, antimicrobial, anxiolytic, neuromodulator, and expectorant, while also highlighting its efficacy as a memory enhancer and antidepressant (Dhingra et al., 2004; El-Saber Batiha et al., 2020; Hasan et al., 2021). Our previous metabolite profiling of *G. glabra* successfully identified well-known bioactive metabolites such as glycyrrhizin, glabridin, and liquiritin, as well as novel metabolites, including quercetin glucosides, coumarin derivatives, β-carotene, and asiatic acid (Karthikkeyan et al., 2020b). The ability of *G. glabra* components, such as glycyrrhizic acid, glabridin, and liquiritin, to cross the blood-brain barrier (Yu et al., 2007; Wang et al., 2015) makes it a potentially promising candidate for the management of neurodegenerative disorders. Understanding the pharmacokinetic profile of *G. glabra* is essential for optimizing its therapeutic efficacy and safety margins. Upon oral administration, the bioactive constituents of this extract exhibit distinct pharmacokinetic profiles; for instance, the primary triterpenoid saponin, glycyrrhizin, is systematically metabolized by the intestinal microbiota into its active metabolite, glycyrrhetinic acid (Asl and Hosseinzadeh, 2008). Furthermore, whole *G. glabra* extracts possess natural bioenhancing properties, with studies demonstrating their capacity to significantly enhance the intestinal absorption and systemic bioavailability of Vitamin B12 (Sharma et al., 2022). While human randomized controlled trials have confirmed the memory and intelligence-enhancing properties of *G. glabra*, suggesting it as a promising candidate for human use, and have demonstrated its safety and efficacy (Sheshagiri et al., 2015), there is a significant knowledge gap regarding its application to AD-specific pathology.

Given the current therapeutic limitations in managing AD, there is a critical and unmet need for disease-modifying therapies that act on the pathophysiology of AD rather than symptoms with no or limited side effects. To address this gap, we tested the neuroprotective effects of *G. glabra* in both the differentiated IMR-32 cell line and the *Drosophila melanogaster* model of Alzheimer’s disease. Our study shows that *G. glabra* provides neuroprotection by modulating cellular pathways involved in oxidative stress, mitochondrial dysfunction, cell cycle regulation, neuronal apoptosis, and dysregulated signaling pathways, including mitogen-activated protein kinase (MEK)-Extracellular Signal-Regulated Kinases 1 and 2 (ERK-1/2) and Protein Kinase B (AKT). This work represents a pioneering endeavor that demonstrates *G. glabra’s* ability to mitigate Alzheimer’s disease. By uncovering the underlying mechanisms of action, this study provides important insights into the therapeutic potential of *G*. *glabra*, paving the way for the development of novel treatment strategies for Alzheimer’s disease.

## Materials and methods

### Reagent procurement

Dimethyl sulfoxide (DMSO, Cat# PCT1303), Propionic acid (Cat# GRM3658), and agar powder (Cat# GRM026) were purchased from HiMedia Laboratories Private Limited, India. Dulbecco’s modified Eagle medium (DMEM, Cat#12100046) high glucose, fetal bovine serum (FBS, Cat# 10270–106), and 100× antibiotic/antimycotic solution (Cat#15240062), Trypsin EDTA solution (Cat#25200-056), Pierce Bicinchoninic Acid (BCA) protein estimation assay kit (Cat# 23225), SuperSignal™ West Atto Ultimate Sensitivity Substrate (Cat# A38555), and FxCycle™ PI/RNase Staining Solution (Cat# F10797) were obtained from Thermo Fisher Scientific USA. PrimeScript RT Reagent Kit (Cat#RR037A) and SYBR Green Master mix (TB Green® Premix ExTaq II (Tli RNase H Plus), Cat#RR820A) were procured from TAKARA BIOINC, Japan. Nitrocellulose membrane (Cat# 1620115) and Clarity ECL Substrate (Cat# 1705061) were purchased from Bio-Rad Laboratories, California, USA. Antibodies were purchased from Cell Signaling Technology, Danvers, USA, and Sigma-Aldrich, St. Louis, USA. Aβ peptide (oligomeric Aβ_42_; Cat# A-1163-2) was procured from rPeptide, USA. Glycyrrhizic acid (Cat# PHR1516), Methanol (Cat# 179337), retinoic acid (Cat# R2625), collagen (Cat#C9791), thiazolyl blue tetrazolium bromide (MTT, Cat# M5655), bisBenzimide H 33,342 (HOECHST, Cat# B2261), 2′,7′-dichlorodihydrofluorescein (DCFDA, Cat# D6883), propidium iodide (Cat# 537059), Triton™ X-100 (X100), Sodium dodecyl sulfate (Cat# L3771), TRIzol™ Reagent (Cat# T9424) and Primers were acquired from Sigma-Aldrich, St. Louis, USA.

### Procurement of *G. glabra* root powder

The present study utilizes root powder of *G. glabra* L (*G. glabra*) commonly known as licorice or Yashtimadhu, belongs to the Fabaceae family. The root powder of *G. glabra* (Batch No. 64) was sourced from the GMP-certified manufacturer SDP Remedies and Research Centre in Puttur, Karnataka, India. Documentation for the root powder with the specimen number SDP/YM/001-2017 is maintained at the Centre. The commercial preparation of *G. glabra* root powder involves drying the roots in a shade net, followed by vacuum drum drying, pulverization of the dried roots, and sieving, resulting in a fine powder with 90% yield. The aqueous extract of *G. glabra* used in this study was previously characterized by our group (Karthikkeyan et al., 2021) using LC-MS/MS. In the present work, the presence of glycyrrhizic acid, the primary bioactive constituent, was confirmed and quantified using High-Performance Liquid Chromatography (HPLC). For cell culture experiments, the aqueous extract of *G. glabra* root was used. 1 g of *G. glabra* root powder was suspended in 10 mL of Milli-Q water (concentration, 0.1 g/mL) and incubated overnight at room temperature with continuous rotation. The extraction mixture was then centrifuged (Neill et al., 1994; Rao and Kisaalita, 2002) at 5,000 g for 10 min. Following SpeedVac drying (Savant, Thermo Fisher Scientific), the *G. glabra* extract was stored at −20 °C. The dried extract was subsequently resuspended in serum-free medium for all cellular treatments.

### Validation of *G. glabra* root powder using reversed-phase high-performance liquid chromatography (RP-HPLC)

The aqueous extract of *G. glabra* was validated by quantifying glycyrrhizic acid (GA) content using reversed-phase high-performance liquid chromatography (RP-HPLC). Briefly, 100 mg of the dry aqueous extract was resuspended in 4 mL of 80% ethanol and subjected to RP-HPLC analysis. The RP-HPLC analysis was carried out using a UHPLC system (Vanquish, Thermo Fisher Scientific, USA). Separation was performed on a reverse-phase column ZORBAX Eclipse Plus C18 (4.6 mm × 150 mm, 5 µm) (Agilent Technologies India Pvt. Ltd.). Solvent A (LC-grade water) and solvent B (50% Acetonitrile) were used as the mobile phase at a flow rate of 0.3 mL/min. Chromatograms were recorded at 270 nm with a run time of 25min throughout the analysis. Solvent B was used as a strong eluent, with its strength increased from 10% to 60% over the first 12 min, then decreased from 60% to 10% over the next 25 min. The glycyrrhizic acid (GA) stock and working solutions were prepared in 80% ethanol. A standard calibration curve was prepared with different concentrations of GA varying from 0.2–1.2 μg/mL. To determine GA concentration in *G. glabra* root powder, the dried and dissolved extract, as mentioned above, was further diluted in methanol and injected into the standardized UHPLC system under the same analytical conditions used to prepare the calibration curve.

### Cell culture and treatment

IMR-32 cells (ATCC® CCL-127™) were procured from the National Centre for Cell Sciences, Pune, India. The cells were maintained in DMEM containing high glucose and L-glutamine, 10% FBS, and 1X antibiotic/antimycotic solution at 37 °C with 5% CO_2_. We differentiated cells on collagen-coated 6-well plates (3 × 10^4^ cells/well) with 10 μM retinoic acid in 2% FBS-supplemented DMEM for 7 days. The resulting differentiated population then underwent a 48-h treatment phase as follows: *G. glabra* aqueous extract, Aβ_42_ treatment (0.5 μM in DMEM) (Modi et al., 2012), and co-treatment with Aβ_42_ and *G. glabra* aqueous extract. The cells not exposed to any treatment served as the control group.

### Cell-cytotoxicity assay

To assess the cytotoxic effects of *G. glabra* root aqueous extract, IMR-32 cells were seeded in a 96-well plate at a density of 5,000 cells per well. These cells were treated with varying extract concentrations (50–2000 μg/mL) for 48 h. After the treatment, MTT dye was added, and the cells were further incubated for 4 h. The formazan crystals formed were dissolved in a 50:50 ethanol/DMSO solution, and the absorbance was measured at 570 nm with background subtraction at 650 nm. Cell viability was then calculated and expressed as a percentage with respect to untreated control cells. The cell cytotoxicity assay was done in biological triplicate.

### Determination of reactive oxygen species (ROS)

As Alzheimer’s disease is associated with increased reactive oxygen species (ROS) production, this study investigated the effect of *G. glabra* on ROS production using the DCFDA method in differentiated IMR-32 cells, as previously described (Karthikkeyan et al., 2021). Seven days of retinoic acid (10 μM) treatment were performed to differentiate IMR-32 cells, which were initially seeded at 3 × 10^4^ cells/well in 1X collagen-treated 6-well plates. After differentiation, the cells were treated with Aβ_42_ (0.5 μM), Aβ_42_ (0.5 μM) + *G. glabra* root aqueous extract (200 μg/mL), *G. glabra* root aqueous extract alone (200 μg/mL), along with the untreated control cells for 48 h. After the treatment, the cells were washed twice with 1X phosphate-buffered saline (PBS) and stained with DCFDA (25 μM) and HOECHST (5 μg/mL) in serum-free media for 20 min in the dark. The cells were imaged using a ZOE™ Fluorescent Cell Imager (BioRad Laboratories, California, USA) at green (excitation 480 nm and emission 517 nm) and blue (excitation 355 nm and emission 433 nm) wavelengths. The images were analyzed using ImageJ (NIH, USA) (Schneider et al., 2012). The intensity of ROS production was then quantified and expressed in fold change for each experimental condition. The assay was performed in biological triplicate.

### Assessment of cell apoptosis using propidium iodide (PI) - HOECHST-staining

IMR32 cells, seeded at 3 × 10^4^ cells/well on 1X collagen-coated plates, underwent a 7-day differentiation with 10 μM retinoic acid before treatment with 0.5 μM Aβ_42_ (0.5 μM), Aβ_42_ (0.5 μM) + *G. glabra* aqueous extract (200 μg/mL), *G. glabra* aqueous extract alone (200 μg/mL) for 48 h, along with the untreated control cells. Treated cells were washed in 1X PBS, followed by a 20-min dark incubation in serum-free medium containing Hoechst (5 μg/mL) for nuclear visualization and PI (20 μg/mL) for cell death assessment (Karthikkeyan et al., 2021). The assay was performed in biological triplicate. ZOE™ Fluorescent Cell Imager (BioRad Laboratories, California, USA) was used to image the cells in red (excitation 556 nm and emission 615 nm) and blue (excitation 355 nm and emission 433 nm) wavelengths, and the images were analyzed using the ImageJ tool, NIH, USA (Schneider et al., 2012). Results for cell viability are reported as fold changes, calculated by comparing treated samples against untreated controls.

### Cell cycle analysis

We initially plated IMR-32 cells at 3 × 10^4^ cells per well using collagen-coated 6-well plates. Following a seven-day differentiation period with 10 μM retinoic acid, the cells were treated for 48 h with Aβ_42_ (0.5 µM), *G. glabra* (200 μg/mL), and Aβ_42_ (0.5 µM) + *G. glabra* (200 μg/mL). Comparisons were made against a control group consisting of untreated cells. The cells were washed with 1X PBS, trypsinized, and washed with 1X PBS before being resuspended in FxCycle™ PI/RNase staining solution (Thermo Fisher Scientific, USA). The cells were then incubated for 20 min in the dark, after which red fluorescence was measured using a Guava® easyCyte Flow Cytometer (EMD Millipore, Massachusetts, USA). FCS Express (version 7) was used to analyze cell cycle data. The assay was performed in biological triplicate.

### Reverse transcription real-time polymerase chain reaction analysis (qRT-PCR)

IMR32 cells were seeded at a density of 3 × 10^4^ cells per well in 1X collagen-coated 6-well plates and were differentiated for 7 days using 10 μM retinoic acid. After differentiation, the cells were treated with Aβ_42_ (0.5 μM), Aβ_42_ (0.5 μM) + *G. glabra* aqueous extract (200 μg/mL), and *G. glabra* aqueous extract alone (200 μg/mL) for 48 h, along with the untreated control cells, respectively. Following a 48-h incubation, cells were rinsed with 1X PBS and harvested via trypsinization. Total RNA isolation was performed using TRI Reagent, as per the standard manufacturer’s guidelines. The concentration and purity of the resulting RNA were then determined with a Colibri microvolume spectrophotometer (Berthold Technologies GmbH and Co. KG, Bad Wildbad, Germany). Reverse transcription was performed to convert RNA to cDNA using the Prime Script 1st strand cDNA synthesis kit (TAKARA BIOINC, Japan). After reverse transcription, qPCR was performed to assess the expression of genes involved in AD, including Acetylcholinesterase (AChE), monoamine oxidase B (MAO-B), beta-site amyloid precursor protein cleaving enzyme 1/Beta-secretase 1 (BACE1), microtubule-associated protein 2 (MAP2), Brain-derived neurotrophic factor (BDNF), and choline acetyltransferase (ChAT). Also, synaptic proteins such as synaptosomal-associated protein of 23 kDa (SNAP23), synaptosomal-associated protein of 25 kDa (SNAP25), synaptophysin, Neuroligin1 (NLGN1), Neuroligin3 (NLGN3), and enolase 2/neuron-specific enolase (ENO2/NSE). qRT-PCR was performed in the CFX96 Touch Real-Time PCR Detection System, BioRad Laboratories, California, USA. RNA expression was quantified by threshold cycle (CT), and relative expression levels were calculated using the ΔΔCT method, with β-actin as the normalization control. The results were expressed as fold change with respect to control cells. All experiments were performed using independent biological triplicates, with each biological sample containing technical triplicates to ensure data accuracy and reproducibility. The details of the primers used in the study are provided in Supplementary Table S1.

### Immunoblotting

Initially, IMR-32 cells were plated into six-well plates (collagen-coated) at a density of 3 × 10^4^ cells per well. Seven days differentiation were carried out using 10 μM retinoic acid. The differentiated cells were subjected to 48 h of treatment with Aβ_42_ (0.5 µM), Aβ_42_ (0.5 µM) + *G. glabra* (200 μg/mL) cotreatment, and *G. glabra* (200 μg/mL) alone. Untreated cells were used as the control group. Following the treatment, the cells were washed twice with 1X PBS and harvested in the cell lysis buffer (2% sodium dodecyl sulfate in 50 mM triethylammonium bicarbonate, with 1 mM sodium orthovanadate, 2.5 mM sodium pyrophosphate, and 1 mM beta-glycerophosphate). Cells were lysed by sonicating them using the Q-Sonica sonicator (Cole-Parmer, India) on ice for three minutes in three cycles at an amplitude of 20%. After centrifuging the lysate at 12,000 × g for 20 min at 4 °C, the protein concentration was estimated using the BCA assay, which used Bovine Serum Albumin (BSA) as a reference standard, and western blotting was carried out as described previously (Amrutha et al., 2025). An equal amount of protein from each of the four conditions was loaded onto sodium dodecyl sulfate-polyacrylamide gel electrophoresis (SDS-PAGE) and resolved. After the SDS-PAGE, proteins were transferred to the nitrocellulose membrane. The membranes were blocked for 1 h in 5% skim milk in 1X phosphate-buffered saline with 0.5% Tween-20 (PBST). Membranes were incubated overnight at 4 °C with primary antibodies at the corresponding dilutions (Supplementary Table S2), which were diluted in 3% BSA in 1X PBST. 1X PBST was used to wash the membranes. Subsequently, the membranes were incubated for 2 h at room temperature with horseradish peroxidase (HRP)-conjugated secondary antibodies in 3% BSA in 1X PBST. The immunoreactive bands were detected with an Enhanced Chemiluminescence reagent on a Fusion© FX chemiluminescence imaging system (VILBER). We quantified band intensities by densitometry using ImageJ (NIH, USA) (Schneider et al., 2012). These values were subsequently normalized to the loading control, and the final results were reported as fold changes relative to the untreated samples. Immunoblotting experiments were performed in three independent biological replicates.

### *Drosophila melanogaster* husbandry

*Drosophila melanogaster* is a valuable model system for studying neurodegenerative disorders (Victor Atoki et al., 2025). To explore Alzheimer’s disease, scientists have developed fly models expressing human amyloid beta proteins in the central nervous system (CNS), which can be effectively used to screen drugs at preliminary levels, providing an array of molecular systems to work on (Prussing et al., 2013). In this study, a transgenic line expressing the Human Aβ_42_ fragment of amyloid beta (A4) precursor protein (APP) under the control of Upstream Activation Sequence (UAS) was used to screen *G. glabra* for mitigating AD.

The wild Canton-S flies are used as control, whereas W1118; Elav-GAL4, and UAS-APP-Aβ_42_ (BL-33774) are the transgenic lines used for expressing the human Aβ_42_ fragment of Amyloid Precursor Protein (APP) in the CNS. The flies were raised in 12 h of light to 12 h of dark conditions. The temperature and humidity were maintained constantly at 23 °C ± 2 °C and 60%–70%. A standard wheat–cream medium (containing semolina, jaggery, agar-agar, and propionic acid) was provided as media for all the strains.

### Treatment with *G. glabra* and toxicity assay

The treatment groups were divided into negative control – wild flies (Canton-S) fed with normal saline; positive control with Aβ_42_ expression (Elav-Gal4xUAS-APP-Aβ_42_) fed with normal saline; flies with Aβ_42_ expression treated with *G. glabra* (200 μg/mL) aqueous extract, and wild flies fed with *G. glabra* aqueous extract (200 μg/mL). The stock solution of *G. glabra* extract was prepared in saline at a concentration of 10 mg/mL. From a 10 mg/mL stock solution of the extract, a 200 μg/mL working solution was prepared by mixing with 5% yeast. Edible food color was added to the working solution to track the consumption of the drug mixed with yeast. The flies were fed for 20 days, during which the media, along with the extract, were changed every 5 days. Thirty flies were added to each vial, and the number of flies was counted every day for 20 days. The toxicity of the drug was analyzed using a survival assay. The number of flies that died each day was noted down. The mortality rate was plotted against the days of treatment and compared with that of controls. The survival assay was performed in two biological duplicates.

### Negative geotaxis/climbing assay

On the 21st day, 10 flies from each group were anesthetized with CO_2_ and transferred to an open-ended tube, with one end closed with parafilm, so the flies were confined within the tube during transfer. After transferring the flies, the remaining end was sealed with parafilm, and a few small holes were poked for the entry of air. The flies were left to recover from anesthesia for about 30 min. It was ensured that the tube was graduated and at least 25 cm long. The climbing assay was carried out by monitoring the flies’ climbing ability. The tube was taped onto a soft surface to ensure the flies were at the bottom (starting) line of the tube. The timer was set to 1 min, and the number of flies that crossed 4 cm–5 cm was recorded. The process was repeated 10 more times for each group. The climbing ability of the flies was plotted as a percentage. The experiment was performed using independent biological duplicates, with each biological sample containing a technical duplicate to ensure data accuracy and reproducibility.

### Sample preparation for immunoblotting

After 20 days of *G. glabra* treatment, around 20 flies from each group were collected into a fresh vial and immobilized by freezing at −20 °C for 30 min to 1 h. After the immobilization, the flies were decapitated under a microscope using 1x PBS. The fly heads were then transferred to 4% Lysis buffer, homogenized using a pellet pestle, and the resulting protein lysates were used for Western blotting experiment as described earlier. The experiment was performed in three independent biological replicates.

### Statistical analysis

Statistical analysis was performed using GraphPad Prism 8 software, and data were presented as the mean ± SEM (standard error of the mean). For *in vitro* studies, experiments were performed using three independent biological replicates (n = 3). Group comparisons were performed using one-way Analysis of Variance (ANOVA) followed by Bonferroni’s *post hoc* test for multiple comparisons. For *in vivo* behavioral studies, experiments were performed with two independent biological replicates (n = 2; with 30 flies per group per replicate). Unpaired Student’s t-test and one-way ANOVA, followed by Dunn’s multiple comparison tests for the survival assay, and Mann-Whitney test for the climbing assay, were performed for *in vivo* behavioral studies. A p-value of ≤0.05 was considered statistically significant.

## Results

### Quantitative identification of glycyrrhizic acid (GA) in *G. glabra* root extract

We previously characterized the aqueous extract of *G. glabra* using LC-MS/MS analysis (Karthikkeyan et al., 2021). For further characterization, we have quantified glycyrrhizic acid in *G. glabra* root extract. A Reversed-Phase High-Performance Liquid Chromatography method was developed to determine and validate GA in *G. glabra* root powder. Good peak resolution was observed with a gradient mobile phase of water: acetonitrile (50:50, v/v). Supplementary Figures S1A,B show optimized chromatograms of the sample and standard. Plotting the calibration curves using peak area versus the concentration of GA standard in the range of 0.2 to 1.2 μg/mL revealed that the calibration plot for the GA standard solution was linear, with a regression equation of y = 561,72x - 1,131.1 Supplementary Figure S1C. The correlation coefficient (R^2^) for the GA standard solution was 0.9963, indicating excellent linearity of the method. The retention time (RT) of the GA standard and sample (*G. glabra* root powder) peaks were found to be at 3.373 and 3.423 min, respectively, and the concentration of GA in our *G. glabra* root powder was calculated to be 182.14 μg/g of powder. This further confirms the authenticity of the *G. glabra* root powder used in the present study.

### Cytotoxicity analysis of *G. glabra* root aqueous extract

To assess the cytotoxicity of *G. glabra* root aqueous extract, IMR-32 cells were treated with various extract concentrations (50 to 2000 μg/mL) for 48 h. The results of our study indicated that the *G. glabra* aqueous extract exhibited no toxicity till 1,500 μg/mL (Supplementary Figure S2). Considering these findings and previous research reports (Karthikkeyan et al., 2020a), we selected a 200 μg/mL concentration for our subsequent cell culture experiments. The treatment doses of Aβ_42_ (Soluble oligomers of Aβ_42_) were taken from the previous study (Modi et al., 2012), i.e., 0.5 μM Aβ_42_.

### *G. glabra* root extract restores ROS production and mitochondrial dysfunction induced by Aβ_42_ in IMR-32 cells and *Drosophila melanogaster* model of Alzheimer’s disease

**FIGURE 1:**
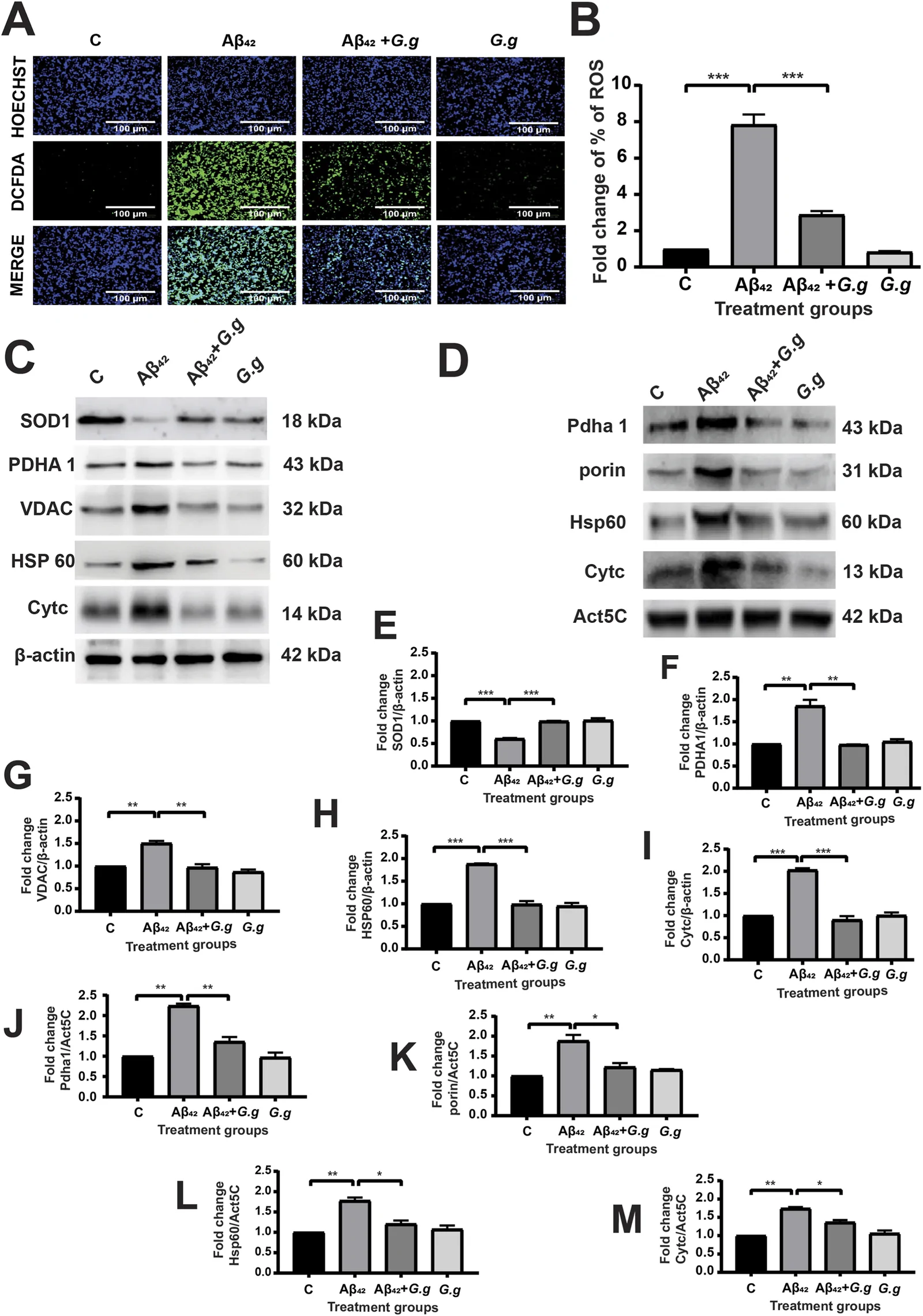
*G. glabra* mitigates Aβ_42_-induced oxidative stress and mitochondrial dysfunction. **(A)** Representative DCFDA fluorescence imaging of ROS generation (green) with Hoechst counterstain (blue) in differentiated IMR-32 cells. **(B)** Quantitative analysis of ROS production in differentiated IMR-32 cells. **(C,D)** Representative Western blots profiling mitochondrial and oxidative stress markers in IMR-32 cells and in *Drosophila melanogaster*, respectively (SOD1, PDHA1/Pdha1, VDAC/Porin, HSP60/Hsp60, Cytochrome C, β-actin/Act5C (homologs of human β-actin) loading control). **(E–M)** Densitometric quantification of the corresponding protein expression levels for **(E–I)** IMR-32 cells and **(J–M)** *Drosophila melanogaster* experimental groups. *p ≤ 0.05, **p ≤ 0.01, ***p ≤ 0.001 (*in vitro* studies n = 3 independent biological replicates; *in vivo* n = 2 independent batches of 30 flies per group). Labels: C: untreated cells/control, Aβ_42_: Aβ_42_ treatment, Aβ_42_ + G.g: Aβ_42_ + *G. glabra* aqueous extract co-treatment, G.g: *G. glabra* aqueous extract treatment, DCFDA: 2′,7′–dichlorofluorescin diacetate, HOECHST: bisBenzimide H 33,342, PDHA1/Pdha1: pyruvate dehydrogenase E1, HSP60/Hsp60: heat shock proteins, VDAC: voltage-dependent anion channel, SOD1: superoxide dismutase type 1, Act5C: Actin 5C, Cytc: Cytochrome c.

To evaluate oxidative stress in differentiated IMR-32 cells, DCFDA staining was employed. This allowed us to measure ROS production, a recognized pathological feature of Alzheimer’s disease. In the Aβ_42_-treated condition, ROS production was significantly elevated to 7.84-fold ±0.54 (p ≤ 0.001) compared to the control. However, co-treatment with *G. glabra* effectively counteracted this increase, reducing ROS production significantly to 2.88-fold ±0.19 (p ≤ 0.001) (Figures 1A,B). These results support the neuroprotective effect of *G. glabra* in the Aβ_42_-induced Alzheimer’s condition. Our findings demonstrate a clear correlation between increased ROS levels and decreased superoxide dismutase type 1 (SOD1) (0.60-fold ±0.01, p ≤ 0.001) expression, alongside elevated pyruvate dehydrogenase E1 (PDHA1) (1.86-fold ±0.13, p ≤ 0.006), voltage-dependent anion channel (VDAC) (1.50-fold ±0.04, p ≤ 0.01), Heat shock protein 60 (Hsp60) (1.88-fold ±0.007, p ≤ 0.001), and cytochrome c levels (2.03-fold ±0.03, p ≤ 0.001), in the Aβ_42_ treated group. However, co-treatment with *G. glabra* significantly reversed these effects, restoring SOD1 activity (0.99-fold ± 0.004, p ≤ 0.001) and normalizing PDHA1 (0.98-fold ± 0.004, p ≤ 0.005), VDAC (0.97-fold ± 0.06, p ≤ 0.008), Hsp60 (0.99-fold ± 0.06, p ≤ 0.001), and cytochrome c expression (0.90-fold ± 0.08, p ≤ 0.001) leading to reduced ROS levels (Figures 1C,E–I). Similarly, *D. melanogaster* with Aβ_42_ overexpression exhibited elevated levels of Pdha1 (2.24-fold ±0.05, p ≤ 0.003), porin (homologs of human VDAC) (1.88-fold ±0.15, p ≤ 0.013), Hsp60 (1.78-fold ±0.07, p ≤ 0.008), and cytochrome c (1.74-fold ±0.03, p ≤ 0.003), indicating mitochondrial impairment. *G. glabra* co-treatment significantly attenuated the elevated protein levels, restoring Pdha1 (1.35-fold ± 0.11, p ≤ 0.011), porin (1.22-fold ±0.10, p ≤ 0.03), Hsp60 (1.20-fold ±0.08, p ≤ 0.02), and cytochrome c (1.36-fold ±0.06, p ≤ 0.04) expression and thereby reducing mitochondrial dysfunction (Figures 1D,J–M). These findings collectively demonstrate the protective efficacy of *G. glabra* against Aβ_42_-induced mitochondrial damage across different biological systems.

### *G. glabra* prevents Aβ_42_-induced apoptosis in differentiated IMR-32 cells and *Drosophila melanogaster*

**FIGURE 2:**
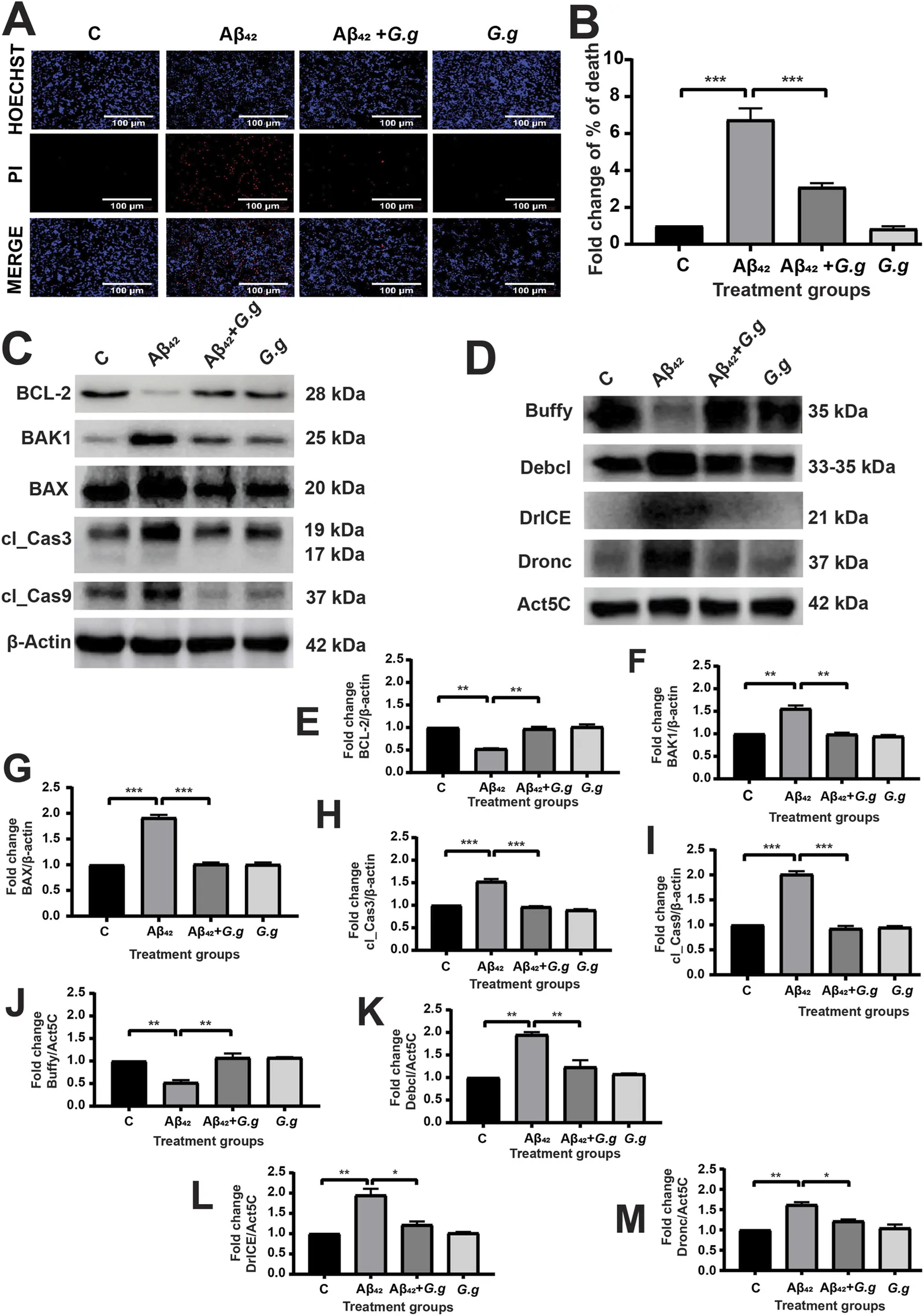
*G. glabra* restores cell apoptosis induced by Aβ_42_: **(A)** Representative live-dead cell staining using propidium iodide (PI; red) and Hoechst counterstain (blue) in differentiated IMR-32 cells. **(B)** Quantitative representation of cell death with respect to the untreated cell control. **(C,D)** Representative Western blot profiling of apoptotic markers in differentiated IMR-32 cells (BCL2, BAK1, BAX, cleaved caspase 3, cleaved caspase 9; β-actin loading control) and *Drosophila melanogaster* (Buffy, Debcl, DrICE, Dronc; Act5C loading control), respectively. **(E–M)** Densitometric quantification of the corresponding apoptotic protein expression levels for **(E–I)** IMR-32 cells and **(J–M)** *Drosophila melanogaster* experimental groups. *p ≤ 0.05, **p ≤ 0.01, ***p ≤ 0.001 (*in vitro* studies, n = 3 independent biological replicates; *in vivo*, n = 2 independent batches of 30 flies per group). Labels: **(C)** untreated cells/control, Aβ_42_: Aβ_42_ treatment, Aβ_42_ + G.g: Aβ_42_ + *G. glabra* aqueous extract co-treatment, G.g: *G. glabra* aqueous extract treatment.

Cell apoptosis was detected using the HOECHST-PI staining assay in differentiated IMR-32 cells (Figures 2A,B). Aβ_42_ treatment significantly increased cell death by 6.75-fold ±0.60 (p ≤ 0.001), while co-treatment with *G. glabra* aqueous extract rescued this effect, reducing cell death to 3.10-fold ±0.20 (p ≤ 0.001). In the Aβ_42_-treated condition, the expression of B-cell lymphoma 2 (BCL2), an anti-apoptotic protein, decreased by 0.53-fold ±0.005 (p ≤ 0.003). In contrast, the expression of BCL2 antagonist/killer 1 (BAK1) (1.56-fold ±0.06, p ≤ 0.002), BCL2-associated X protein (BAX) (1.92-fold ±0.04, p ≤ 0.001), cleaved caspase-3 (1.53-fold ±0.04, p ≤ 0.001), and cleaved caspase-9 (2.01-fold ±0.05, p ≤ 0.001), proteins promoting apoptosis (pro-apoptotic proteins), are increased. This imbalance favored apoptosis of neurons and may contribute to the neuronal loss observed in AD. Interestingly, co-treatment with Aβ_42_ and *G. glabra* effectively reversed these abnormal conditions, restoring the expression levels of BCL2 (0.97-fold ±0.04, p ≤ 0.03), BAK1 (0.99-fold ±0.03, p ≤ 0.002), BAX (1.01-fold ±0.02, p ≤ 0.001), cleaved caspase-3 (0.96-fold ±0.009, p ≤ 0.001), and cleaved caspase-9 (0.93-fold ±0.04, p ≤ 0.001) to normal levels in diffrentiated IMR-32 cells, as shown in Figures 2C,E–I.

Interestingly, a similar pattern was observed in *Drosophila melanogaster*, where Buffy (homologs of human BCL2) expression was also reduced (0.52 ± 0.05, p ≤ 0.01), while Death executioner Bcl2 homologue (Debcl) (homologs of human BAK1) (1.95 ± 0.05, p ≤ 0.005), *Drosophila* Interleukin-1β-Converting Enzyme (DrICE) (homologs of human cleaved caspase-3) (1.95 ± 0.15, p ≤ 0.009), and Death regulator Nedd2-like caspase (Dronc) (homologs of human cleaved caspase-9) (1.62 ± 0.06, p ≤ 0.007) were elevated in transgenic flies with Aβ_42_ overexpression. Similarly, co-treatment with *G. glabra* restored the expression of Buffy (1.07 ± 0.09, p ≤ 0.01), Debcl (1.23 ± 0.14, p ≤ 0.01), DrICE (1.22 ± 0.08, p ≤ 0.02), and Dronc (1.22 ± 0.05, p ≤ 0.03) in transgenic flies (Figures 2D,J–M). Overall, these findings demonstrate that *G. glabra* effectively blocks Aβ_42_-induced apoptotic signaling in both *in vitro* and *in vivo* models.

### The *G. glabra* restores Aβ_42_-induced cell cycle re-entry

**FIGURE 3:**
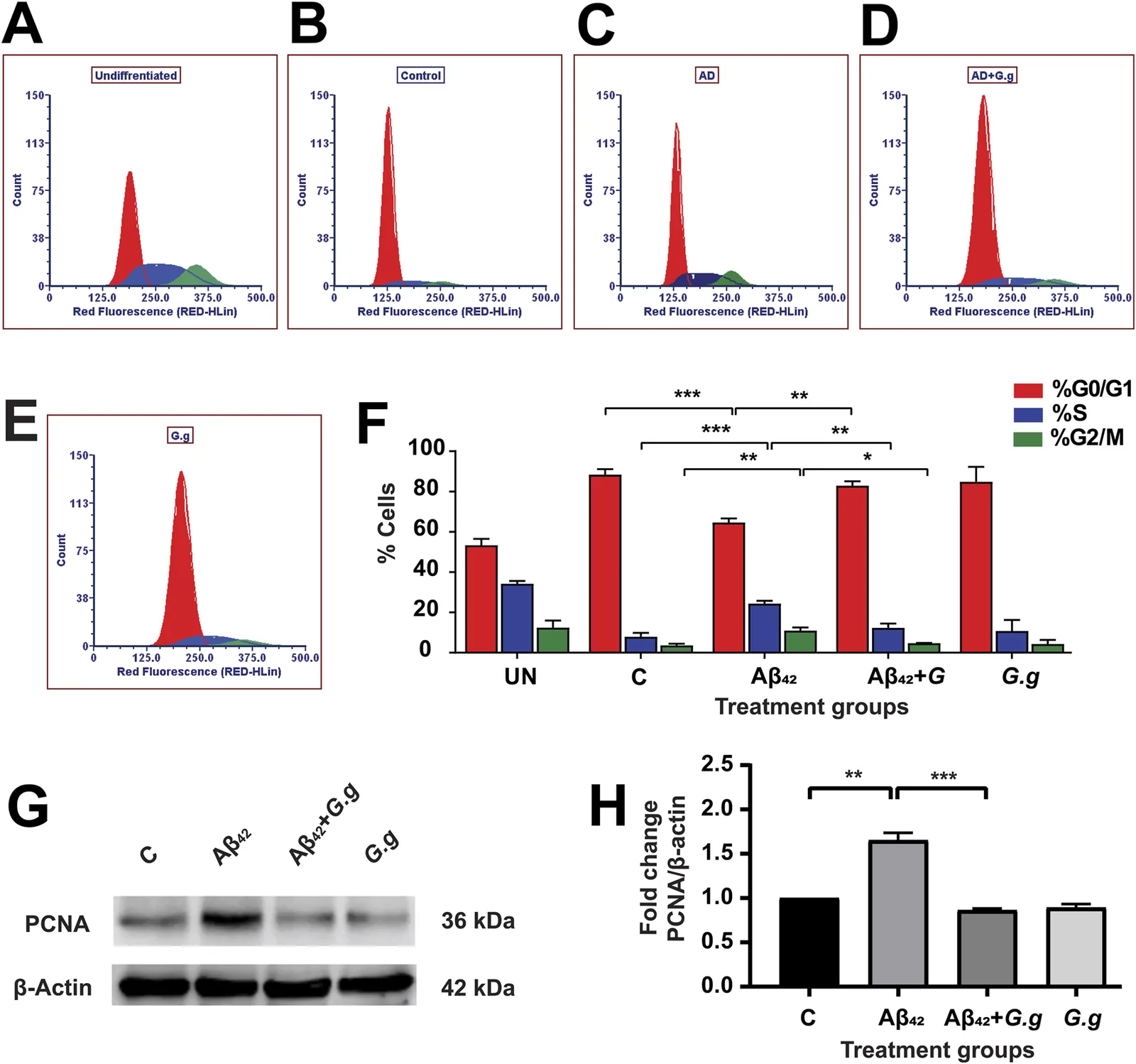
*G. glabra* restores Aβ_42_-induced cell cycle re-entry. **(A)** Undifferentiated IMR32 cells, **(B)** Differentiated and untreated cells control, **(C)** Aβ_42_ (0.5 µM), **(D)** Aβ_42_ (0.5 µM) with *G. glabra* (200 μg/mL) co-treatment, **(E)** *G. glabra* alone (200 μg/mL), and **(F)** Graphical representation of cell population across different phases of cell cycle, **(G)** Immunoblot analysis of PCNA, and **(H)** densitometry graph of PCNA. Differentiated and untreated cells were taken as a control. *p ≤ 0.05, **p ≤ 0.01, ***p ≤ 0.001, n = 3. Labels: C: untreated cells, Aβ_42_: Aβ_42_ treatment, Aβ_42_ + G.g: Aβ_42_ + *G. glabra* aqueous extract co-treatment, G.g: *G. glabra* aqueous extract treatment, PCNA: Proliferating Cell Nuclear Antigen.

Cell cycle analysis (Figures 3A–F) revealed that the differentiated IMR-32 control cells predominantly resided in the G0/G1 phase (88.40%). Treatment with *G. glabra* also resulted in a significant number of cells in the G0/G1 phase (84.87%), while S-phase and G2/M-phase cells were notably lower in both the control (S-phase: 7.95%, G2/M-phase: 3.63%) and *G. glabra* (S-phase: 10.81%, G2/M-phase: 4.31%) treatment groups. Compared to the control, Aβ_42_ treatment led to a significant increase in cells in the S-phase (24.37%) and G2/M-phase (10.97%). However, co-treatment with Aβ_42_ and *G. glabra* significantly reversed the cells from the S-phase (12.35%) and G2/M (4.72%) back to the G0/G1 phase (82.92%). Undifferentiated IMR-32 cells exhibited a distinct cell cycle distribution compared to the differentiated cells, with 53.34% in G0/G1, 34.20% in S, and 12.45% in G2/M phase. Immunoblotting analysis using the S-phase marker proliferating cell nuclear antigen (PCNA) confirmed these findings, showing upregulation of PCNA in the Aβ_42_ treatment group to 1.65-fold ±0.08 (p ≤ 0.003) compared to the control. This upregulation was restored to 0.86-fold ±0.01 (p ≤ 0.001) by *G. glabra* co-treatment Figures 3G,H. These results demonstrate that *G. glabra* prevents Aβ_42_-induced aberrant cell cycle re-entry.

### *G. glabra* rescues Aβ_42_-induced messenger ribonucleic acid (mRNA) alterations associated with Alzheimer’s disease in differentiated IMR-32 cells

**FIGURE 4:**
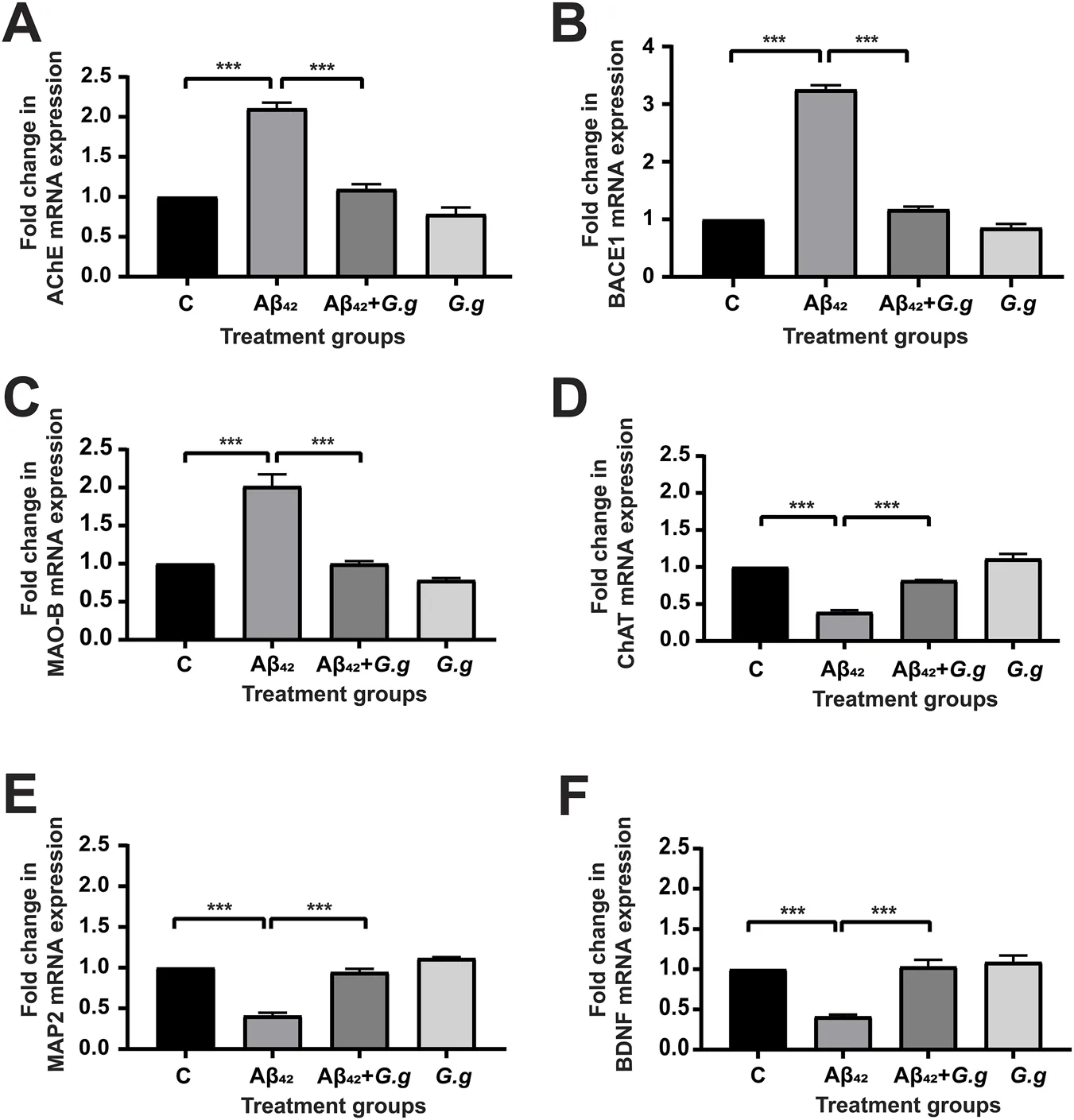
*G. glabra* mitigates the Aβ_42_-induced alterations in gene expression. Gene expression analysis of **(A)** AChE, **(B)** BACE1, **(C)** MAO-B, **(D)** ChAT, **(E)** MAP2, and **(F)** BDNF in the differentiated IMR-32 cells treated with an aqueous extract of *G. glabra* in the Alzheimer’s disease model as analyzed using qRT-PCR. *p ≤ 0.05, **p ≤ 0.01, ***p ≤ 0.001, n = 3. Labels: C: untreated cells, Aβ_42_: Aβ_42_ treatment, Aβ_42_ + G.g: Aβ_42_ + *G. glabra* aqueous extract co-treatment, G.g: *G. glabra* aqueous extract treatment, AChE: Acetylcholinesterase, BACE1:beta-site amyloid precursor protein cleaving enzyme 1/Beta-secretase 1, MAO-B: monoamine oxidase B, ChAT: choline acetyltransferase, MAP2: microtubule-associated protein 2 and BDNF:- Brain-derived neurotrophic factor.

qRT-PCR analysis revealed increased mRNA expression of AChE (2.10-fold ± 0.07, p ≤ 0.001), BACE1 (3.25-fold ± 0.07, p ≤ 0.001), and MAO-B (2.01-fold ± 0.15, p ≤ 0.001), while ChAT (0.39-fold ± 0.02, p ≤ 0.001), MAP2 (0.41-fold ± 0.03, p ≤ 0.001), and BDNF (0.41-fold ± 0.02, p ≤ 0.001) gene expression was decreased in Aβ_42_-treated conditions. *G. glabra* co-treatment effectively normalized these mRNA levels, restoring AChE (1.09-fold ± 0.06, p ≤ 0.001), BACE1 (1.17-fold ± 0.04, p ≤ 0.001), and MAO-B (1.00-fold ± 0.03, p ≤ 0.001) expression to control levels and significantly increasing ChAT (0.81-fold ± 0.007, p ≤ 0.001), MAP2 (0.94-fold ± 0.03, p ≤ 0.001), and BDNF (1.03-fold ± 0.08, p ≤ 0.001) expression (Figure 4).

**FIGURE 5:**
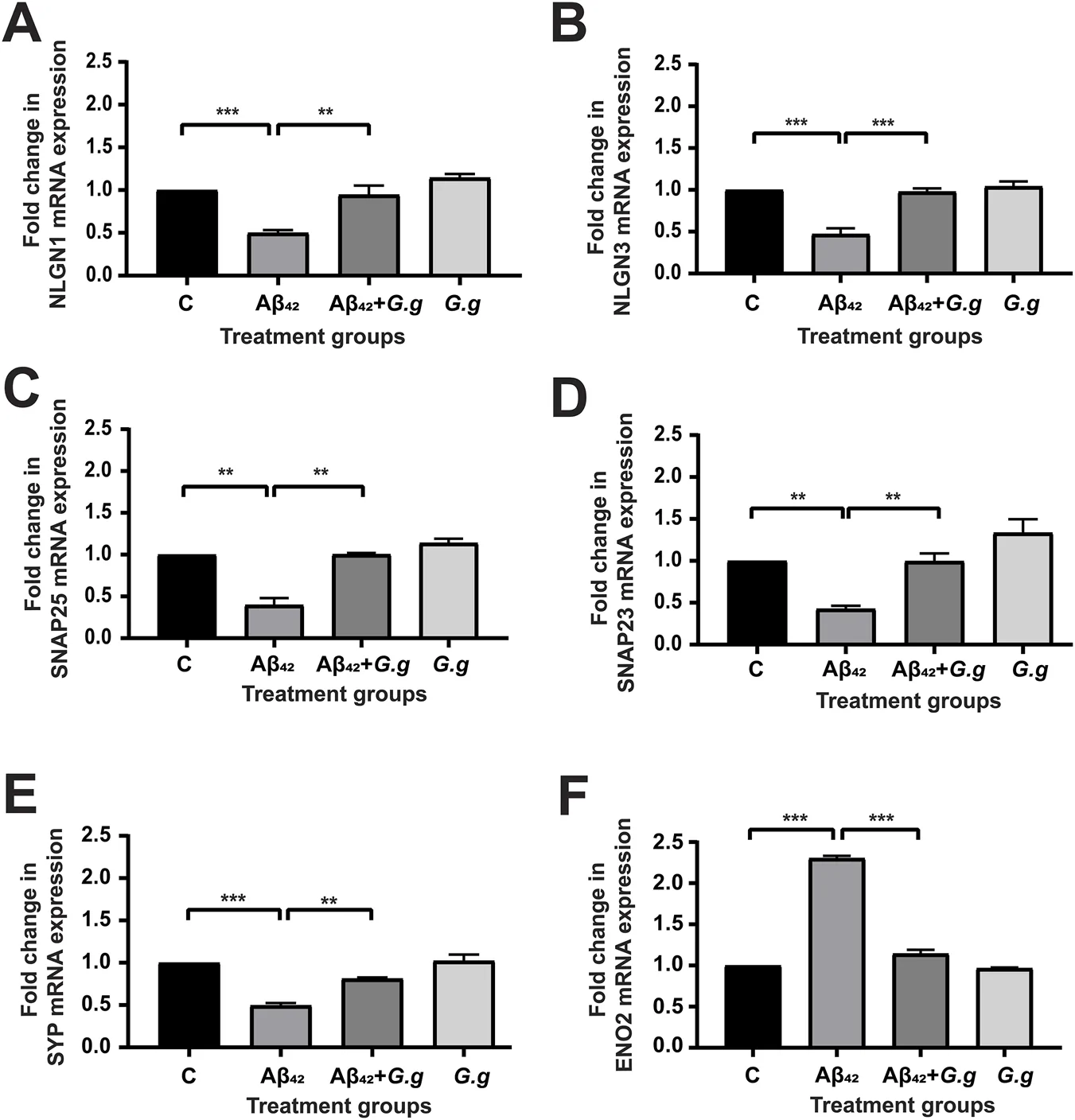
*G. glabra* reverses the Aβ_42_-mediated dysregulation of gene expression of synaptic proteins. Gene expression analysis of **(A)** NLGN1, **(B)** NLGN3, **(C)** SNAP25, **(D)** SNAP23, **(E)** SYP, and **(F)** ENO2 in the differentiated IMR-32 cells treated with an aqueous extract of *G. glabra* in the Alzheimer’s disease model as analyzed using qRT-PCR. *p ≤ 0.05, **p ≤ 0.01, ***p ≤ 0.001, n = 3. Labels: C: untreated cells, Aβ_42_: Aβ_42_ treatment, Aβ_42_ + G.g: Aβ_42_ + *G. glabra* aqueous extract co-treatment, G.g: *G. glabra* aqueous extract treatment, NLGN1: Neuroligin1, NLGN3: Neuroligin 3, SNAP25: synaptosomal-associated protein of 25kDa, SNAP23: synaptosomal-associated protein of 23kDa, SYP: synaptophysin, and ENO2: enolase 2/neuron-specific enolase (NSE).

Furthermore, Aβ_42_ treatment resulted in a significant decrease in mRNA levels of the synaptic proteins NLGN1 (0.50-fold ± 0.03, p ≤ 0.001), NLGN3 (0.47-fold ± 0.07, p ≤ 0.001), SNAP25 (0.39-fold ± 0.08, p ≤ 0.005), SNAP23 (0.42-fold ± 0.03, p ≤ 0.01), and Synaptophysin (0.49-fold ± 0.02, p ≤ 0.001). Conversely, ENO2 mRNA levels were elevated by 2.30-fold ± 0.02 (p ≤ 0.001) in Aβ_42_-treated conditions. *G. glabra* co-treatment effectively reversed these effects, restoring NLGN1 (0.94-fold ± 0.10, p ≤ 0.003), NLGN3 (0.97-fold ± 0.03, p ≤ 0.001), SNAP25 (1.00-fold ± 0.01, p ≤ 0.005), SNAP23 (0.99-fold ± 0.09, p ≤ 0.01), and Synaptophysin (0.81-fold ± 0.01, p ≤ 0.003) mRNA to normal levels and significantly reducing the elevated ENO2 mRNA expression by 1.14-fold ± 0.04 (p ≤ 0.001) (Figure 5). Collectively, these data show that *G. glabra* reduces core Alzheimer’s protein pathology while maintaining synaptic integrity.

### *G. glabra* restores aberrant alterations in proteins involved in Alzheimer’s disease pathology and synapse

**FIGURE 6:**
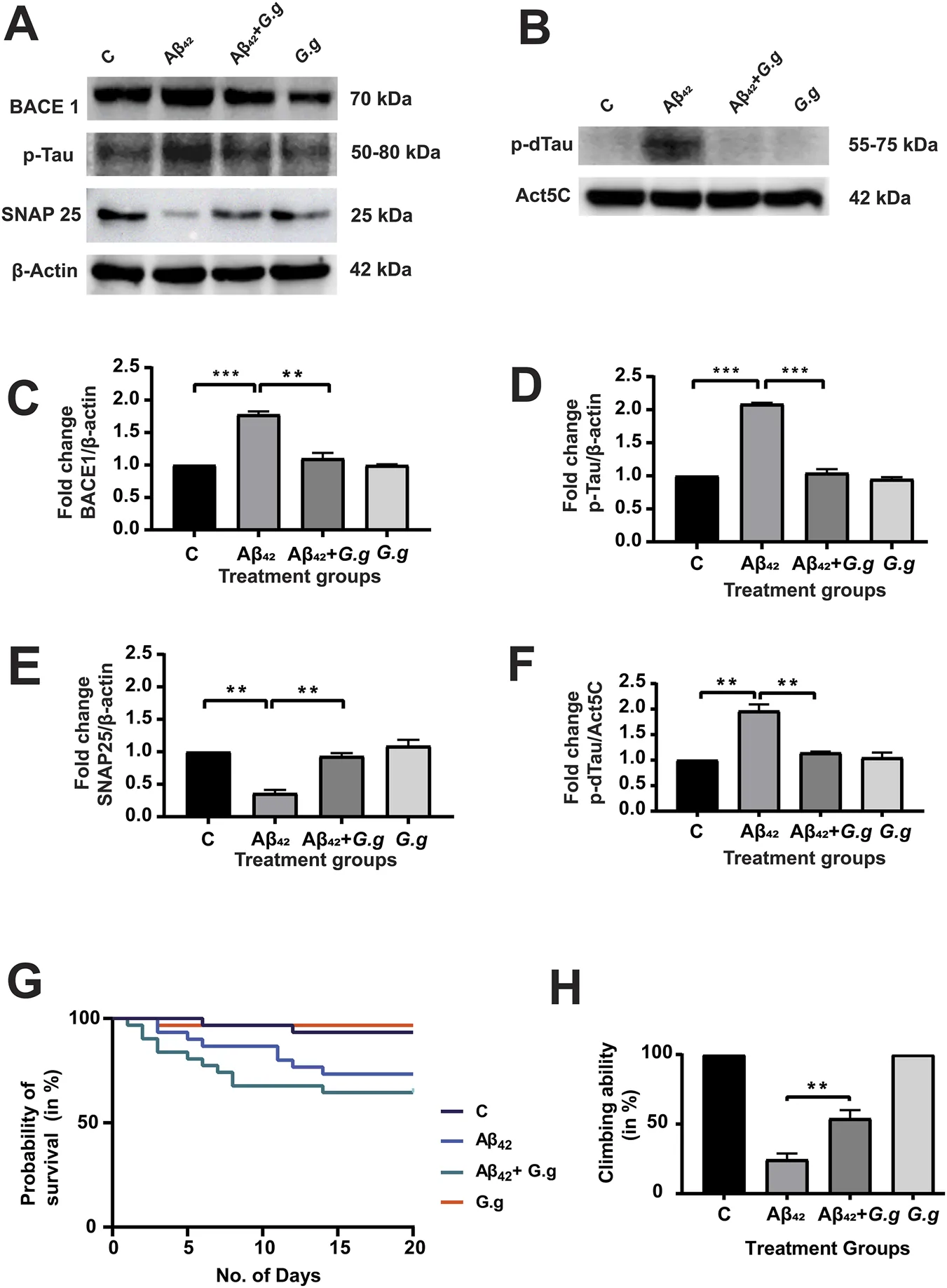
*G. glabra* restores Aβ_42_ induced aberrant protein expression and synaptic function in Alzheimer’s Disease: **(A)** immunoblot analysis of BACE1, p-Tau, and SNAP25 in diffrntiated IMR-32 cells treated with *G. glabra*, **(B)** immunoblot analysis of p-dTau in Aβ_42_-overexpressing *Drosophila melanogaster* transgenic flies fed with *G. glabra*, **(C–F)** Densitometric quantification of the corresponding protein expression levels for **(C–E)** diffrentiated IMR-32 cells and **(F)** *Drosophila melanogaster* experimental groups. **(G)** Representation of the probability of survival (in %, in Y-axis) of control (Canton-S), Aβ_42_-induced *Drosophila melanogaster* transgenic fly line, *G. glabra*-treated Aβ_42_-induced *Drosophila melanogaster* transgenic flies, and control (Canton-S) treated with *G. glabra*. The duration of treatment, measured in days, is represented along the X-axis, and **(H)** Representation of the % of climbing ability of the flies in all four conditions. *p ≤ 0.05, **p ≤ 0.01, ***p ≤ 0.001 (*in vitro* studies n = 3 independent biological replicates; *in vivo* n = 2 independent batches of 30 flies per group). Labels: C: control, Aβ_42_: Aβ_42_-induced AD model, Aβ_42_ + G.g: Aβ_42_-induced AD model + *G. glabra* aqueous extract co-treatment (200 µg/mL), G.g: *G. glabra* aqueous extract treatment (200 μg/mL), BACE1: beta-site amyloid precursor protein cleaving enzyme 1, p-Tau: phosphorylated Tau, SNAP25: synaptosomal-associated protein of 25 kDa, and p-dTau: *Drosophila* Tau protein.

The two primary hallmarks of AD pathology are the accumulation of amyloid plaques and neurofibrillary tangles (NFTs) in the brain. BACE1 protein cleaves the APP at the β-site, releasing amyloid-β, and the hyperphosphorylation of the Tau protein (microtubule-associated protein tau (MAPT) forms p-Tau. Our western blotting results demonstrated that Aβ_42_ treatment significantly increased BACE1 (1.78-fold ±0.04, p ≤ 0.001) and p-Tau (2.08-fold ±0.01, p ≤ 0.001) proteins in differentiated IMR-32 cells Figures 6A,C,D. At the same time, *G. glabra* co-treatment substantially reduced BACE1 (1.10-fold ±0.08, p ≤ 0.003) and p-Tau (1.04-fold ±0.05, p ≤ 0.001).

Similarly, in *Drosophila melanogaster*, p-dTau expression was elevated to 1.96-fold ±0.13 (p ≤ 0.006), Figures 6B,F, which was further significantly restored to 1.14-fold ±0.02 (p ≤ 0.01) upon *G. glabra* co-treatment, thereby preventing the accumulation of amyloid plaques and Tau tangles. SNAP25 plays a crucial role in synaptic function and exocytosis, which was found to be decreased in Aβ_42_-treated differentiated IMR-32 cells to 0.36-fold ±0.05 (p ≤ 0.008). The expression of SNAP25 was remarkably reversed and restored to normal by *G. glabra* co-treatment (0.93-fold ±0.04, p ≤ 0.01), depicted in Figures 6A,E. Collectively, these data highlight the neuroprotective capacity of *G. glabra* to reverse biochemical hallmarks of Alzheimer’s disease and safeguard synaptic integrity.

### *G. glabra* restores behavioral deficits associated with Alzheimer’s disease in *Drosophila melanogaster*

#### Administration of *G. glabra* does not induce toxicity in *Drosophila melanogaster*

The survival assay was done to test the toxicity of the *G. glabra* treatment on *Drosophila melanogaster*. The flies were treated with 200 μg/mL of *G. glabra* for a duration of 20 days. Wild-type flies showed no significant difference in their survival after the *G. glabra* treatment when compared to normal saline fed wild-type flies. The Aβ_42_-induced *Drosophila* flies showed decreased probability of survival against wild-type flies, irrespective of the treatment. High mortality in the Aβ_42_-induced *Drosophila* line is a strong indicator that the human Aβ_42_ gene is being expressed (Prussing et al., 2013). However, the treatment of *G. glabra* had no significant toxic effect on the survival of Aβ_42_-induced *Drosophila* flies when compared to the saline-treated Aβ_42_ overexpressed group (Figure 6G).

### *G. glabra* improved locomotor performance impaired by Aβ_42_ in *Drosophila melanogaster*

The motor symptoms are often associated with AD and contribute to increased mortality and can serve as predictive markers for AD in older adults (Andrade-Guerrero et al., 2024). The AD model of *Drosophila melanogaster* has been previously reported to show challenged locomotory activities due to Aβ_42_-induced toxicity (Singh et al., 2014; Deolankar et al., 2023), so we checked the effect of *G. glabra* extract feeding using a climbing assay on Aβ_42_-overexpressing flies after a treatment period of 20 days. The results showed no significant difference in the climbing ability of Canton-S flies fed on normal saline in media compared to those treated with *G. glabra*. However, there was a significant improvement (p ≤ 0.012) in the climbing ability of *G. glabra*-treated Aβ_42_-induced *Drosophila* flies when compared to the Aβ_42_-induced flies that did not receive any treatment. This indicates an enhancement in locomotor activity due to the *G. glabra* treatment (Figure 6H).

### *G. glabra* prevents Aβ_42_-induced hyperactivation of signaling molecules associated with Alzheimer’s disease

**FIGURE 7:**
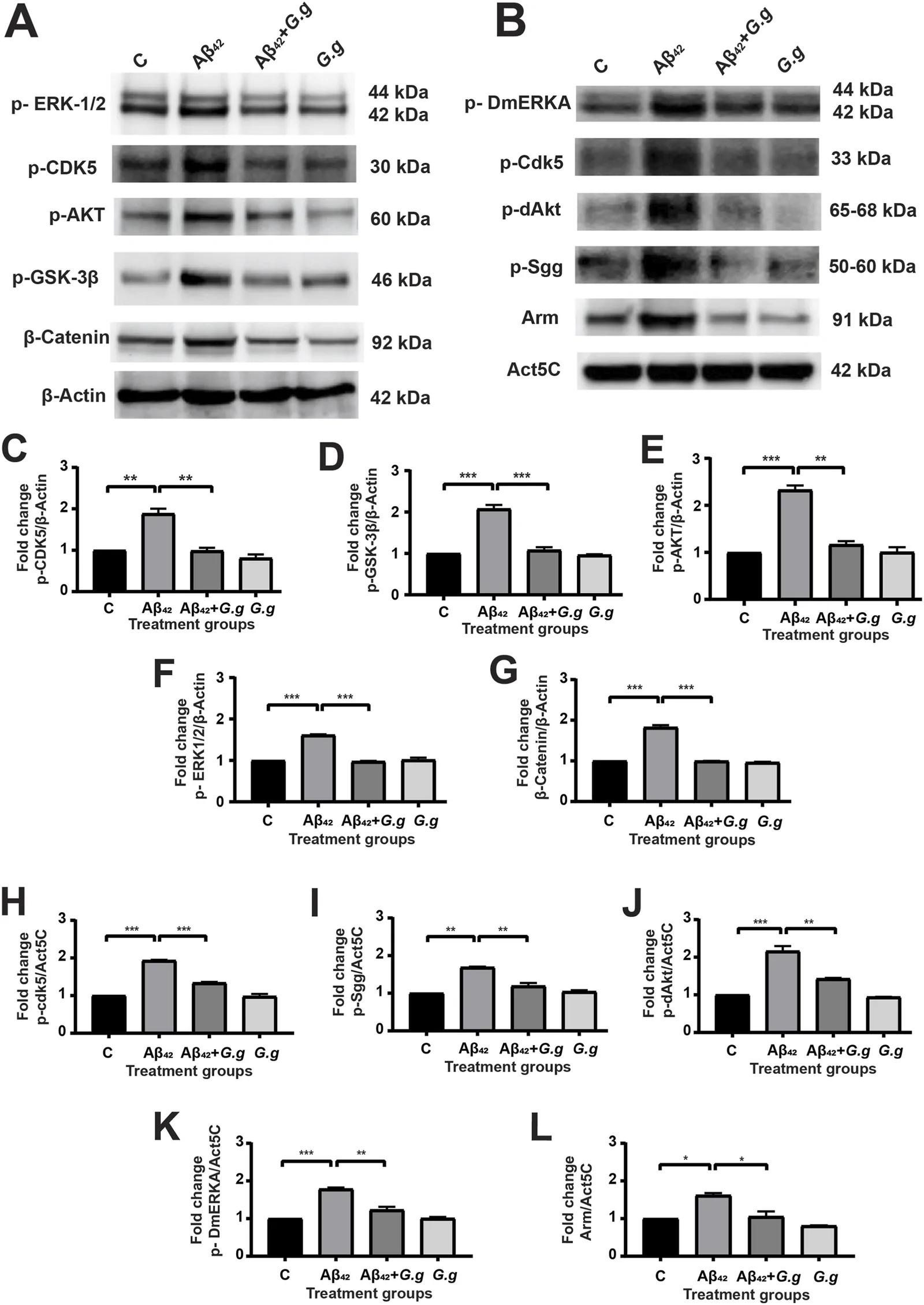
*G. glabra* inhibits hyperactivation of signaling molecules induced by Aβ_42_ in both *in vitro* and *in vivo* model of Alzheimer’s Disease: **(A)** immunoblot analysis of p-CDK5, p-GSK3β, p-AKT, p-ERK-1/2, and β-Catenin in diffrentiated IMR-32 cells treated with *G. glabra*, **(B)** immunoblot analysis of p-Cdk5, p-Sgg, p-dAkt, p-DmERKA and, Armadillo (Arm) in Aβ_42_ overexpressed *Drosophila melanogaster* transgenic flies fed with *G. glabra*, **(C)** densitometry graph of p-CDK5, **(D)** densitometry graph of p-GSK3β, **(E)** densitometry graph of p-AKT, **(F)** densitometry graph of p-ERK-1/2, **(G)** densitometry graph of β-Catenin in IMR-32 cells treated with *G. glabra*, **(H)** densitometry graph of p-Cdk5, **(I)** densitometry graph of p-Sgg, **(J)** densitometry graph of p-dAkt, **(K)** densitometry graph of p-DmERKA and, **(L)** densitometry graph of Arm in *Drosophila melanogaster* treated with *G. glabra*. *p ≤ 0.05, **p ≤ 0.01, ***p ≤ 0.001 (*in vitro* studies n = 3 independent biological replicates; *in vivo* studies n = 2 independent batches of 30 flies per group). Labels: C: control, Aβ42: Aβ42-induced AD model, Aβ42 + G.g: Aβ42-induced AD model + *G. glabra* aqueous extract co-treatment (200 μg/mL), G:g: *G. glabra* aqueous extract treatment (200 μg/mL).

Treatment with Aβ_42_ significantly promoted the phosphorylation of cyclin-dependent kinase 5 (CDK5), resulting in a 1.88-fold ± 0.11 increase (p ≤ 0.008), glycogen synthase kinase-3 beta (GSK3β) (2.07-fold ± 0.09, p ≤ 0.001), AKT (2.33-fold ± 0.09, p ≤ 0.001), and ERK-1/2 (1.61-fold ± 0.01, p ≤ 0.001) proteins compared to control. Concomitantly, β-catenin levels were significantly elevated in Aβ_42_-treated cells (1.82-fold ± 0.05, p ≤ 0.001). Co-treatment with *G. glabra* effectively reversed the hyperphosphorylation of CDK5 (0.99-fold ± 0.07, p ≤ 0.008), GSK3β (1.08-fold ± 0.06, p ≤ 0.001), AKT (1.17-fold ± 0.06, p ≤ 0.002), and ERK1/2 (0.97-fold ± 0.01, p ≤ 0.001) proteins, induced by Aβ_42_ treatment in IMR-32 cells. Additionally, β-catenin levels were restored to 0.99-fold ± 0.003 (p ≤ 0.001) in *G. glabra* co-treated cells (Figures 7A,C–G).

The same trend was observed in *Drosophila melanogaster,* Aβ_42_ overexpression also led to elevated phosphorylation of cdk5 (1.93-fold ± 0.02, p ≤ 0.001), Shaggy (Sgg) (homologs of human GSK3β) (1.69-fold ± 0.01, p ≤ 0.002), dAkt (2.16-fold ± 0.13, p ≤ 0.001), and DmERKA ((homologs of human ERK2) (1.78-fold ± 0.04, p ≤ 0.001), along with an increase in Armadillo (Arm) (homologs of human β-catenin) (1.61-fold ± 0.05, p ≤ 0.02). *G. glabra* co-treatment also significantly restored the hyperphosphorylation of Cdk5 (1.33-fold ± 0.02, p ≤ 0.001), Sgg (1.19-fold ± 0.08, p ≤ 0.007), dAkt (1.42-fold ± 0.02, p ≤ 0.009), and DmERKA (1.23-fold ±0.08, p ≤ 0.007). Furthermore, Armadillo levels were restored to 1.05-fold ±0.13 (p ≤ 0.03), as depicted in Figures 7B,H,I. These findings suggest that Aβ_42_ treatment induces a cascade of protein phosphorylation events and β-catenin hyperactivation, which may contribute to the pathological processes underlying Alzheimer’s disease. *G. glabra*, through its ability to restore these proteins to normal levels, demonstrates potential neuroprotective effects.

## Discussion

The predominant pathological characteristics of AD are senile plaques primarily composed of amyloid β peptides, and neurofibrillary tangles. In AD, brain atrophy due to neuronal loss is a prominent pathological feature (Erekat, 2022). Aβ_42_ deposition is believed to precede other pathological events, like NFT formation and neuronal loss. The transition from Aβ_42_ accumulation to neurodegeneration is driven by amyloidogenic processing of the amyloid precursor protein, in which BACE1 initiates the production of Aβ_42_ peptides, which aggregate into neuritic plaques and contribute to synaptic dysfunction by modulating synaptic vesicle release (Das et al., 2021). Excessive Aβ_42_ accumulation leads to various cytotoxic effects, including oxidative stress, ionic imbalance (Canevari et al., 2004), and the hyperactivation of kinases such as GSK3β, CDK5, Protein Kinase A (PKA), Fyn kinase, and microtubule affinity-regulating kinase (MARK), which in turn leads to Tau hyperphosphorylation, NFT formation, and synaptic degeneration (Chin et al., 2000; Wu et al., 2025). This ultimately activates cell death cascades in neurons (Niikura et al., 2006), contributing to memory impairment and cognitive deficits (Serrano-Pozo et al., 2011).

The cognitive and memory impairments observed in AD are profoundly driven by cholinergic dysfunction, which is characterized by a loss of cholinergic neurons and a subsequent reduction in ChAT, the enzyme responsible for acetylcholine (ACh) biosynthesis, and increased expression of AChE, leading to decreased ACh levels. In AD, ChAT activity is significantly reduced in the cerebral cortex, hippocampus, and temporal and frontal lobes, often correlating with dementia severity (Lopez and DeKosky, 2003). AChE plays a crucial role in the rapid hydrolysis of ACh in the central and peripheral nervous system, and research indicates that Aβ_42_ and p-Tau further increase AChE expression by elevating intracellular calcium levels (Sberna et al., 1997). Furthermore, MAO-B, which is upregulated in AD, is linked to disease etiology by potentially inducing Aβ synthesis and impairing cognitive performance. Both AChE and MAO-B can affect APP processing and Aβ production by modulating γ-secretase levels (Campanari et al., 2014; Schedin-Weiss et al., 2017).

Given that these interconnected enzymatic and neurotransmitter disruptions are widely reported to be clinically altered in AD, we selected these specific target proteins and genes to evaluate whether *G. glabra* could modulate this pathological phenotype and restore these disease-induced alterations to physiological control levels. In the present study, *G. glabra* co-treatment restored elevated levels of BACE1 and p-Tau, restored cholinergic neurotransmission, and prevented the loss of neurons. These neuroprotective effects are likely attributed to the high levels of phenolics, flavonoids, and antioxidants in *G. glabra* (Martins et al., 2015; Pastorino et al., 2018). Phenolics and flavonoids are known for their anti-aging and neuroprotective activities, primarily due to their potent antioxidant properties, which allow them to scavenge harmful free radicals in the brain (Ullah et al., 2020; Rojas-Garcia et al., 2023). Furthermore, they help prevent protein aggregation by inhibiting proteolytic pathways, such as the ubiquitin-proteasome system, and downregulate oxidative stress and the release of pro-inflammatory cytokines, thereby controlling the apoptotic response (Naoi et al., 2019).

This antioxidant capacity is linked to the stabilization of oxidative stress and mitochondrial dysfunction, a prominent and early feature of AD (D'Alessandro et al., 2025). Aβ_42_-induced neurotoxicity triggers endoplasmic reticulum (ER) stress and nitric oxide stress, generating ROS that impair mitochondrial ATP production, leading to oxidative damage and subsequent programmed neuronal death (D'Alessandro et al., 2025). We observed a clear correlation between increased ROS generation and decreased SOD1 activity, along with elevated PDHA1 levels, in the presence of Aβ_42_. Disruptions in PDHA1 are associated with ER stress preceding mitochondrial impairment (Yang et al., 2018). *G. glabra* co-treatment significantly reversed these effects, restoring ROS production, SOD1 activity, and normalizing PDHA1 expression in both differentiated IMR32 cells and the *Drosophila* model.

The severe oxidative stress and metabolic imbalance in AD directly initiate downstream mitochondrial apoptotic pathways by compromising mitochondrial membrane integrity. In AD, VDAC (part of the mitochondrial permeability transition pore) facilitates the release of cytochrome c into the cytosol, a critical player in the apoptotic pathway (McCommis and Baines, 2012). Additionally, phosphorylated Tau has been shown to interact with VDAC1 and Drp1, impairing mitochondrial function and axonal transport (Rajmohan and Reddy, 2017). Furthermore, Hsp60 levels are increased in AD, potentially as a neuroprotective mechanism, yet Hsp60 is also involved in the mitochondrial mislocalization of APP and Aβ, contributing to disease progression (Walls et al., 2012; Marino Gammazza et al., 2022). We found that increases in VDAC, cytochrome c, and Hsp60 were all restored by *G. glabra* co-treatment, suggesting that its neuroprotective role is mediated by modulating mitochondrial function and protein aggregation, and by preventing the release of apoptotic factors.

Apoptosis is regulated by proteins such as Bcl2, BAX, BAK, and caspases, with Bcl2 preventing and BAX/BAK promoting apoptosis (Kitamura et al., 1998; Youle and Strasser, 2008). Aβ_42_ downregulates BCL2 and upregulates BAX in human neurons (Paradis et al., 1996). Activation of the intrinsic apoptotic pathway, triggered by events such as DNA damage, leads to the release of cytochrome c from mitochondria. Cytochrome c interacts with apoptotic protease-activating factor 1 (APAF-1, a cytosolic protein), forming a complex that activates caspase-9, a key player in apoptosis (Boatright et al., 2003). Increased activated caspase 9 levels are observed in the hippocampus of AD patients. Activated caspase 9 initiates a cascade of caspase activation, including downstream effector caspases such as caspases 3, 6, and 7, which degrade intracellular proteins (Deveraux et al., 1997). Elevated caspase 3 activity in AD neurons is believed to be a hallmark of increased cell death (Su et al., 2001). Our findings of live-dead assay and western blotting experiments demonstrate that co-treatment with *G. glabra* restores these altered apoptotic proteins to normal levels in both *in vitro* and *in vivo* studies, suggesting its potential to prevent neuronal death by protecting against mitochondrial dysfunction and the subsequent release of apoptotic factors.

In addition to apoptotic signaling, progressive neuronal loss is highly influenced by aberrant cell-cycle re-entry. Pathological evidence suggests that normal brain neurons are typically quiescent in the G0 phase. In contrast, AD neurons exhibit signs of cell cycling, which precedes plaque formation and neurofibrillary tangles (NFT), leading to neurodegeneration (Lee et al., 2009; Bonda et al., 2010). Studies have found elevated levels of DNA replication markers, such as chromosome-maintenance protein MCM2, in AD neurons, indicating a transition through the S-phase (Bonda et al., 2009). Overexpression of cyclins and PCNA in AD brains may contribute to neuronal death (Bonda et al., 2010). In our study, *G. glabra* significantly reversed this cell cycle re-entry to normal levels, as evidenced by reduced PCNA expression, thereby preventing neuronal apoptosis.

This stabilization of the cell cycle is not an isolated event, but is fundamentally driven by the homeostatic modulation of upstream intracellular kinase cascades. This stabilization is strongly supported by the modulation of the ERK-1/2/CDK5/GSK3β and PI3K/AKT/GSK3β pathways (Amrutha et al., 2026). Increased ERK-1/2 phosphorylation correlates with elevated APP levels, potentially accelerating AD pathology (Ephrame et al., 2023) and upregulates CDK5 expression (Lee and Kim, 2004). CDK5 and GSK3β hyperactivation promote Tau hyperphosphorylation, Aβ_42_ production, and neurodegeneration (Hooper et al., 2008; Ao et al., 2022). Additionally, GSK3β phosphorylation enhances β-catenin levels (Sun, 2009). The GSK3β/β-catenin signaling pathway is associated with synaptic plasticity and cognitive function (Zhang et al., 2021). Dysfunction in the PI3K/AKT pathway increases GSK3β activity, promoting Tau hyperphosphorylation (Lee et al., 2003; Kitagishi et al., 2014). Additionally, CDK5 can promote neuronal survival by regulating AKT activity via the neuregulin/PI3K signaling pathway (Li et al., 2003). Both the CDK5/p35/p25 complexes and the PI3K/AKT/GSK3β pathway have been shown to modulate Tau hyperphosphorylation in the hippocampus of aged male mice (Neri-Gomez et al., 2017). Notably, in our study, we observed dysregulated phosphorylation of these signaling molecules, and their activity was restored to normal levels by co-treatment with *G. glabra* in both *in vitro* and *in vivo* studies. This signaling restoration directly impacts p-Tau hyperphosphorylation and MAP2 levels. Several studies have reported that MAP2 levels are typically decreased in Alzheimer’s disease, particularly in the dentate gyrus (Li et al., 2008). MAP2 caps Tau fibrils and inhibits their aggregation (Holden et al., 2023). By restoring these proteins and kinases, *G. glabra* effectively intercepts the molecular drivers of neurofibrillary tangles and amyloidogenic processing.

The preservation of these intracellular kinase pathways and cytoskeletal proteins helps maintain synaptic integrity and behavioral function. Synapse loss and damage across brain regions are critical features of AD, contributing to the onset and progression of behavioral and physiological symptoms (Jeong, 2017). Aβ_42_ impairs synaptic vesicle trafficking and reduces presynaptic proteins, including SNAP23, SNAP25, synaptophysin, and synaptotagmin, in AD patients (John and Reddy, 2021; Kunii et al., 2021). SNAP23 plays a crucial role in synaptic plasticity and spatial memory (Huang et al., 2023). Reduced SNAP25 is associated with neuronal degeneration (Hoerder-Suabedissen et al., 2019). Synaptophysin, a presynaptic vesicle protein involved in exocytosis, endocytosis, and synapse formation, is also reduced in AD (Tarsa and Goda, 2002; John and Reddy, 2021) and serves as a marker for disease progression and correlates with cognitive decline.

Furthermore, postsynaptic Neuroligins (NLGN1 and NLGN3) are essential for synaptic transmission, plasticity, synapse formation, and maintenance (Jedlicka et al., 2015). NLGN1 is crucial for long-term memory (Kim et al., 2008), and NLGN3, a CDK5 substrate regulated by CDK5-mediated phosphorylation, is involved in synapse assembly at both excitatory and inhibitory synapses, is implicated in neurodevelopmental disorders, and is decreased by Aβ oligomers (Camporesi et al., 2021; Jeong et al., 2023).

The functional capacity of these preserved synapses is further reinforced by the normalization of metabolic markers and the active upregulation of neurotrophic support. In our study, we observed elevated levels of Neuron-specific enolase, suggesting altered neuronal metabolism. It plays regulatory roles beyond glycolysis and gluconeogenesis, including in hypoxia, ischemia, and AD (Schmidt et al., 2014). Crucially, our results demonstrated that *G. glabra* treatment mitigates the Aβ_42_-induced downregulation of these key pre- and post-synaptic proteins and their gene expression levels. While direct electrophysiological or functional synaptic transmission assays were not performed in this study, the restoration of these structural synaptic markers is likely driven by the upregulation of BDNF, a key regulator of neurogenesis, survival, synaptic plasticity, and neuronal differentiation (Numakawa and Odaka, 2021). Given that reduced BDNF expression is a hallmark of AD pathogenesis that correlates with cognitive decline in both clinical and animal models (Jiao et al., 2016; Du et al., 2018), the ability of *G. glabra* to restore BDNF expression profiles is a vital component of its neuroprotective effects. This molecular effect aligns with the known potential of flavonoids, such as quercetin, to promote BDNF expression and improve cognitive function in AD models (Karimipour et al., 2019).

The translation from molecular stabilization to physiological and behavioral recovery is illustrated by the reversal of behavioral deficits in the *Drosophila* model. As locomotor deficiency is strongly linked to neurodegenerative progression, leading to direct consequences for impaired motor function (Cunningham et al., 2018; Ruitenberg, 2024). The improved climbing ability observed in *G. glabra*-treated flies provides phenotypic validation that correlates with the normalized expression of the evaluated synaptic markers and BDNF. Collectively, while future electrophysiological studies are warranted to confirm the absolute functional synaptic transmission, our findings demonstrate that *G. glabra* is a promising therapeutic candidate that stabilizes vital intracellular signaling pathways and synaptic protein networks, leading to tangible improvements in cognitive behavior.

The neuroprotective potential observed in this study aligns with previous literature demonstrating the therapeutic capabilities of individual constituents of *G. glabra* across various neuropathological models. Specifically, glabridin has demonstrated the ability to reverse amnesia induced *in vivo* and suppress acetylcholinesterase activity (Cui et al., 2008). At the molecular level, glycyrrhizin (glycyrrhizic acid) exhibits potent anti-inflammatory and anti-excitotoxic properties, mitigating microglial activation, astrogliosis, and proinflammatory signaling across ischemic, status epilepticus, and excitotoxic models (Kim et al., 2012; Luo et al., 2013; Gonzalez-Reyes et al., 2016). Additionally, liquiritigenin blocks glutamate-induced apoptosis by suppressing Ca (2+) influx, ROS production, and Mitogen-Activated Protein Kinase (MAPK) pathway activation while preserving mitochondrial BCL2/BAX balance (Yang et al., 2013), which is complemented by liquiritin’s antioxidant upregulation and modulation of AKT/GSK3β and ERK-1/2 signaling (Teng et al., 2014; Nakatani et al., 2017). Collectively, the neuroprotective potential presented in our study may be attributed to the concurrent presence of these diverse bioactive constituents in *G. glabra* extract.

**FIGURE 8:**
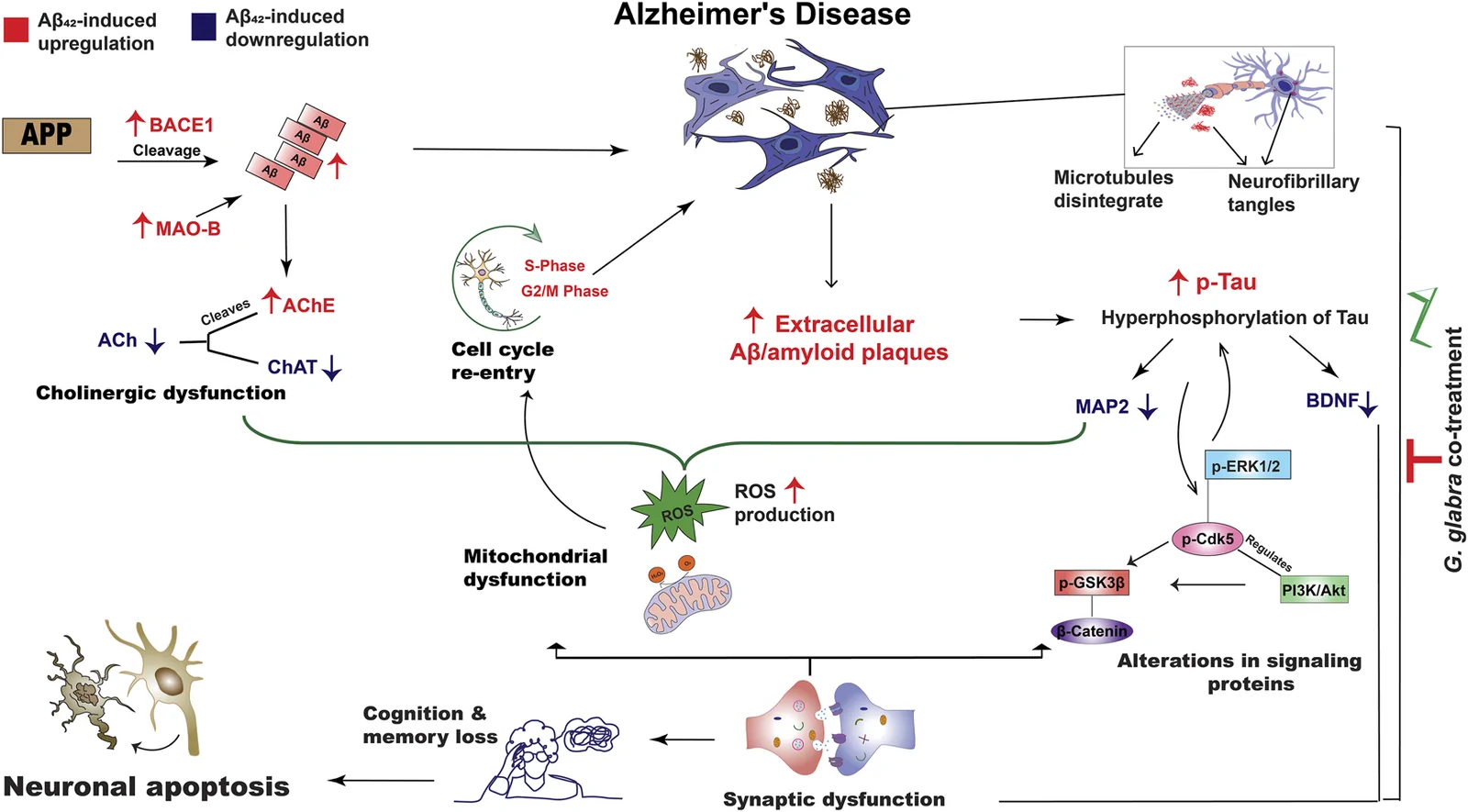
Schematic representation that summarizes the key findings from both the *in vitro* and *in vivo* experiments presented in this study. Labels: APP: amyloid precursor protein, BACE1: beta-site amyloid precursor protein cleaving enzyme 1/Beta-secretase 1, MAO-B: monoamine oxidase B, Aβ: amyloid beta, ACh: acetylcholine, AChE: acetylcholinesterase, ChAT: choline acetyltransferase, MAP2: microtubule-associated protein 2, BDNF: brain-derived neurotrophic factor, ROS: reactive oxygen species, p-ERK-1/2: phosphorylated extracellular signal-regulated kinases 1 and 2, p-CDK5: phosphorylated cyclin-dependent kinase 5, p-ββ: Phospho-glycogen synthase kinase 3 beta, PI3K/AKT: phosphatidylinositol 3-kinase/Protein Kinase B, *G. glabra*: *Glycyrrhiza glabra*.

In conclusion, through a comprehensive analysis using ROS assays, live-dead staining, qPCR, cell cycle assays, Western blotting, and behavioral studies, this research provides a baseline evaluation of how *G. glabra* root extract alters well-reported pathological markers associated with Alzheimer’s disease, both *in vitro* and *in vivo*. Our findings document changes in oxidative stress markers, cell-cycle balance, apoptotic pathways, and the expression of key neuronal proteins, with Figure 8 providing a visual summary of these observed alterations. However, the precise upstream signaling mechanisms through which these pathological cascades progress in Alzheimer’s disease, and how the extract fundamentally intervenes, remain to be determined. Ultimately, these descriptive results provide a solid foundation for future mechanistic studies and the ongoing development of plant-based interventions for Alzheimer’s disease.

### Limitations and future perspectives

In the present study, we utilize differentiated IMR-32 cells as a model system. While the human neuroblastoma IMR-32 cell line is a valuable, established *in vitro* system for investigating Aβ_42_-induced toxicity, APP processing, and human-derived neuronal signaling profiles (Neill et al., 1994; Rao and Kisaalita, 2002), it has inherent limitations as a monoculture model. Specifically, isolated cell lines cannot fully replicate the complex, multicellular microenvironment of the Alzheimer’s disease brain, which involves intricate interactions among neurons, astrocytes, and microglia, as well as systemic factors such as blood-brain barrier (BBB) permeability. Furthermore, questions regarding the systemic bioavailability and metabolic processing of whole-plant extracts require validation beyond a controlled culture environment, and additional validation is needed to assess mechanistic specificity. Therefore, future *in vivo* investigations are required to fully evaluate the therapeutic efficacy, BBB penetration, systemic bioavailability, hepatic metabolism, safety, and clinical translational potential of *G. glabra* extracts in Alzheimer’s pathology.

Although this study provides valuable baseline insights into the neuroprotective efficacy of *G. glabra* root extract, it also possesses certain limitations that must be acknowledged. Our exploration of oxidative stress parameters was focused, and assessing a broader panel of biomarkers, including lipid peroxidation, protein carbonylation, and glutathione status, is essential to comprehensively differentiate direct antioxidant activity from broader cytoprotective mechanisms. While the targeted kinase pathways are heavily cross-linked in Alzheimer’s pathology, further functional experiments utilizing pathway-specific inhibitors or network analysis are required to definitively map direct signaling crosstalk. Finally, evaluating key inflammatory signaling molecules and cytokines remains a crucial next step to establish the extract’s anti-inflammatory potential. Given current structural and resource constraints, these comprehensive mechanistic pathways, time-course evaluations, and multi-marker screens will be actively considered and prioritized in our future *in vitro* and *in vivo* validation studies.

## Data Availability

The original contributions presented in the study are included in the article/Supplementary Material, further inquiries can be directed to the corresponding author.

## Supplementary material

SUPPLEMENTARY FIGURE S1
**(A)** HPLC chromatogram of glycyrrhizic acid standard (200 ng/mL), **(B)** *G. glabra* plant crude extracts, and **(C)** Calibration curve of glycyrrhizic acid standard, ranging from 200 ng/mL to 1200 ng/mL.

SUPPLEMENTARY FIGURE S2 Cytotoxicity analysis of different concentrations of *G. glabra* aqueous extract.

SUPPLEMENTARY TABLE S1 List of primers and their sequences used in the study.

SUPPLEMENTARY TABLE S2 List of antibodies used in the study.

## Glossary

**Aβ**_**42**_: Amyloid Beta 42
AD: Alzheimer’s disease
ADR: adverse drug reactions
APAF-1: Apoptotic Protease Activating Factor 1
APP: amyloid precursor protein
ANOVA: Analysis of Variance
ACh: acetylcholine
AChE: Acetylcholinesterase
ATP: Adenosine triphosphate
AKT: Protein Kinase B
BCA: bicinchoninic acid
Bcl2: B-cell lymphoma 2
Bak1: Bcl2 antagonist/killer 1
BAX: Bcl2-associated X protein
BBB: Blood-brain barrier
BSA: bovine serum albumin
BACE1: beta-site amyloid precursor protein cleaving enzyme 1/Beta-secretase 1
BDNF: Brain-derived neurotrophic factor
ChAT: choline acetyltransferase
CDK5: cyclin-dependent kinase 5
cDNA: complementary deoxyribonucleic acid
CSF: cerebrospinal fluid
CT: computed tomography
**CO**_**2**_: Carbon dioxide
COX-2: Cyclooxygenase-2
Ca (2+): Calcium
Debcl: Death executioner Bcl2
DMSO: dimethyl sulfoxide
DMEM: Dulbecco’s modified Eagle medium
DNA: deoxyribonucleic acid
DrICE: *Drosophila* Interleukin-1β-Converting Enzyme
Dronc: Death regulator Nedd2-like caspase
Drp1: dynamin-related protein 1
ECL: enhanced chemiluminescence
EDTA: ethylenediaminetetraacetic acid
ERK-1/2: Extracellular signal-Regulated Kinases 1 and 2
ER: endoplasmic reticulum
FC reagent: Folin–Ciocalteu reagent
FDA: food and drug administration
GABA: gamma-aminobutyric acid
p-GSK3β: Phospho-glycogen synthase kinase 3 beta
HOECHST: bisBenzimide H 33342
HRP: horseradish peroxidase
Hsp60: Heat shock protein 60
HMGB1: High mobility group box 1
iNOS: Inducible Nitric Oxide Synthase
MAO-B: monoamine oxidase B
MAP2: microtubule-associated protein 2
MARK: microtubule affinity-regulating kinase
MAPK: Mitogen-Activated Protein Kinase
MCM2: minichromosome maintenance complex component 2
MeCN: Methyl Cyanide/Acetonitrile
MEK: mitogen-activated protein kinase kinase
MPTP: mitochondrial permeability transition pore
MRI: magnetic resonance imaging
mRNA: messenger ribonucleic acid
MTT: thiazolyl blue tetrazolium bromide
NIH: national institutes of health
NMDA: N-methyl-D-aspartate
NMDAR: N-methyl-D-aspartate receptor
NPPs: natural plant products
PBS: phosphate-buffered saline
PCNA: proliferating cell nuclear antigen
PI3K: phosphatidylinositol 3-kinase
PNS: peripheral nervous system
PKA: Protein Kinase A
qRT-PCR: quantitative reverse transcription polymerase chain reaction
RNA: ribonucleic acid
RNase: ribonuclease
RP-HPLC: Reversed-Phase High-Performance Liquid Chromatography
ROS: reactive oxygen species
SDS-PAGE: sodium dodecyl sulfate-polyacrylamide gel electrophoresis
TNF-α: Tumor Necrosis Factor-alpha
VAChT: Vesicular acetylcholine transporter.

## References

[B1] Amrutha S. Abhinand C. S. Upadhyay S. S. Parvaje R. Prasad T. S. K. Modi P. K. (2025). Network pharmacology and metabolomics analysis of Tinospora cordifolia reveals BACE1 and MAOB as potential therapeutic targets for neuroprotection in alzheimer's disease. Sci. Rep. 15 (1), 8103. 10.1038/s41598-025-92756-5 40057579 PMC11890609

[B2] Amrutha S. Prasad T. S. K. Modi P. K. (2026). Glycyrrhizic acid attenuates Abeta(42)-Induced neurodegeneration through coordinated regulation of oxidative stress, synaptic markers, and key alzheimer's signaling pathways. Cells 15 (5), 436. 10.3390/cells15050436 41827870 PMC12984613

[B3] Andrade-Guerrero J. Martinez-Orozco H. Villegas-Rojas M. M. Santiago-Balmaseda A. Delgado-Minjares K. M. Perez-Segura I. (2024). Alzheimer's disease: understanding motor impairments. Brain Sci. 14 (11), 1054. 10.3390/brainsci14111054 39595817 PMC11592238

[B4] Ao C. Li C. Chen J. Tan J. Zeng L. (2022). The role of Cdk5 in neurological disorders. Front. Cell. Neurosci. 16, 951202. 10.3389/fncel.2022.951202 35966199 PMC9368323

[B5] Arnsten A. F. T. Datta D. Del Tredici K. Braak H. (2021). Hypothesis: tau pathology is an initiating factor in sporadic alzheimer's disease. Alzheimers Dement. 17 (1), 115–124. 10.1002/alz.12192 33075193 PMC7983919

[B6] Asl M. N. Hosseinzadeh H. (2008). Review of pharmacological effects of Glycyrrhiza sp. and its bioactive compounds. Phytother. Res. 22 (6), 709–724. 10.1002/ptr.2362 18446848 PMC7167813

[B7] Boatright K. M. Renatus M. Scott F. L. Sperandio S. Shin H. Pedersen I. M. (2003). A unified model for apical caspase activation. Mol. Cell. 11 (2), 529–541. 10.1016/s1097-2765(03)00051-0 12620239

[B8] Bonda D. J. Evans T. A. Santocanale C. Llosa J. C. Vina J. Bajic V. (2009). Evidence for the progression through S-phase in the ectopic cell cycle re-entry of neurons in Alzheimer disease. Aging (Albany NY) 1 (4), 382–388. 10.18632/aging.100044 19946466 PMC2783633

[B9] Bonda D. J. Lee H. P. Kudo W. Zhu X. Smith M. A. Lee H. G. (2010). Pathological implications of cell cycle re-entry in Alzheimer disease. Expert Rev. Mol. Med. 12, e19. 10.1017/S146239941000150X 20584423 PMC2922901

[B10] Campanari M. L. Garcia-Ayllon M. S. Belbin O. Galceran J. Lleo A. Saez-Valero J. (2014). Acetylcholinesterase modulates presenilin-1 levels and gamma-secretase activity. J. Alzheimers Dis. 41 (3), 911–924. 10.3233/JAD-140426 24699279

[B11] Camporesi E. Lashley T. Gobom J. Lantero-Rodriguez J. Hansson O. Zetterberg H. (2021). Neuroligin-1 in brain and CSF of neurodegenerative disorders: investigation for synaptic biomarkers. Acta Neuropathol. Commun. 9 (1), 19. 10.1186/s40478-021-01119-4 33522967 PMC7852195

[B12] Canevari L. Abramov A. Y. Duchen M. R. (2004). Toxicity of amyloid beta peptide: tales of calcium, mitochondria, and oxidative stress. Neurochem. Res. 29 (3), 637–650. 10.1023/b:nere.0000014834.06405.af 15038611

[B13] Chin J. Y. Knowles R. B. Schneider A. Drewes G. Mandelkow E. M. Hyman B. T. (2000). Microtubule-affinity regulating kinase (MARK) is tightly associated with neurofibrillary tangles in Alzheimer brain: a fluorescence resonance energy transfer study. J. Neuropathol. Exp. Neurol. 59 (11), 966–971. 10.1093/jnen/59.11.966 11089574

[B14] Collaborators G. B. D. D. F. Steinmetz J. D. Vollset S. E. Fukutaki K. Chalek J. Abd-Allah F. (2022). Estimation of the global prevalence of dementia in 2019 and forecasted prevalence in 2050: an analysis for the global burden of disease study 2019. Lancet Public Health 7 (2), e105–e125. 10.1016/S2468-2667(21)00249-8 34998485 PMC8810394

[B15] Cui Y. M. Ao M. Z. Li W. Yu L. J. (2008). Effect of glabridin from Glycyrrhiza glabra on learning and memory in mice. Planta Med. 74 (4), 377–380. 10.1055/s-2008-1034319 18484526

[B16] Cunningham P. C. Waldeck K. Ganetzky B. Babcock D. T. (2018). Neurodegeneration and locomotor dysfunction in Drosophila scarlet mutants. J. Cell. Sci. 131 (18), jcs216697. 10.1242/jcs.216697 30154211 PMC6176922

[B17] D'Alessandro M. C. B. Kanaan S. Geller M. Pratico D. Daher J. P. L. (2025). Mitochondrial dysfunction in Alzheimer's disease. Ageing Res. Rev. 107, 102713. 10.1016/j.arr.2025.102713 40023293

[B18] Das B. Singh N. Yao A. Y. Zhou J. He W. Hu X. (2021). BACE1 controls synaptic function through modulating release of synaptic vesicles. Mol. Psychiatry 26 (11), 6394–6410. 10.1038/s41380-021-01166-2 34158621 PMC8760050

[B19] Deolankar S. C. Najar M. A. Ramesh P. Kanichery A. Kudva A. K. Raghu S. V. (2023). Discovery of molecular networks of neuroprotection conferred by Brahmi extract in Abeta(42)-Induced toxicity model of *Drosophila melanogaster* using a quantitative proteomic approach. Mol. Neurobiol. 60 (1), 303–316. 10.1007/s12035-022-03066-0 36261695

[B20] DeTure M. A. Dickson D. W. (2019). The neuropathological diagnosis of Alzheimer's disease. Mol. Neurodegener. 14 (1), 32. 10.1186/s13024-019-0333-5 31375134 PMC6679484

[B21] Deveraux Q. L. Takahashi R. Salvesen G. S. Reed J. C. (1997). X-linked IAP is a direct inhibitor of cell-death proteases. Nature 388 (6639), 300–304. 10.1038/40901 9230442

[B22] Dhingra D. Parle M. Kulkarni S. K. (2004). Memory enhancing activity of Glycyrrhiza glabra in mice. J. Ethnopharmacol. 91 (2-3), 361–365. 10.1016/j.jep.2004.01.016 15120462

[B23] Du Y. Wu H. T. Qin X. Y. Cao C. Liu Y. Cao Z. Z. (2018). Postmortem brain, cerebrospinal fluid, and blood neurotrophic factor levels in Alzheimer's disease: a systematic review and meta-analysis. J. Mol. Neurosci. 65 (3), 289–300. 10.1007/s12031-018-1100-8 29956088

[B24] El-Saber Batiha G. Magdy Beshbishy A. El-Mleeh A. Abdel-Daim M. M. Prasad Devkota H. (2020). Traditional uses, bioactive chemical constituents, and pharmacological and toxicological activities of Glycyrrhiza glabra L. (Fabaceae). Biomolecules 10 (3), 352. 10.3390/biom10030352 32106571 PMC7175350

[B25] Ephrame S. J. Cork G. K. Marshall V. Johnston M. A. Shawa J. Alghusen I. (2023). O-GlcNAcylation regulates extracellular signal-regulated kinase (ERK) activation in Alzheimer's disease. Front. Aging Neurosci. 15, 1155630. 10.3389/fnagi.2023.1155630 37469955 PMC10352608

[B26] Erekat N. S. (2022). Apoptosis and its therapeutic implications in neurodegenerative diseases. Clin. Anat. 35 (1), 65–78. 10.1002/ca.23792 34558138

[B27] Gonzalez-Reyes S. Santillan-Cigales J. J. Jimenez-Osorio A. S. Pedraza-Chaverri J. Guevara-Guzman R. (2016). Glycyrrhizin ameliorates oxidative stress and inflammation in hippocampus and olfactory bulb in lithium/pilocarpine-induced status epilepticus in rats. Epilepsy Res. 126, 126–133. 10.1016/j.eplepsyres.2016.07.007 27490898

[B28] Gupta D. K. Chaudhuri A. (2025). Lecanemab and Donanemab in early alzheimer’s disease: a systematic review of clinical, biomarker, and safety outcomes. CHRISMED J. Health Res. 12 (3), 167–174. 10.4103/cjhr.cjhr_48_25

[B29] Hansen R. A. Gartlehner G. Webb A. P. Morgan L. C. Moore C. G. Jonas D. E. (2008). Efficacy and safety of donepezil, galantamine, and rivastigmine for the treatment of Alzheimer's disease: a systematic review and meta-analysis. Clin. Interv. Aging 3 (2), 211–225. 18686744 PMC2546466

[B30] Hasan M. K. Ara I. Mondal M. S. A. Kabir Y. (2021). Phytochemistry, pharmacological activity, and potential health benefits of Gly cyrrhiza glabra. Heliyon 7 (6), e07240. 10.1016/j.heliyon.2021.e07240 34189299 PMC8220166

[B31] Hoerder-Suabedissen A. Korrell K. V. Hayashi S. Jeans A. Ramirez D. M. O. Grant E. (2019). Cell-Specific loss of SNAP25 from cortical projection neurons allows normal development but causes subsequent neurodegeneration. Cereb. Cortex 29 (5), 2148–2159. 10.1093/cercor/bhy127 29850799 PMC6754517

[B32] Holden M. R. Krzesinski B. J. Weismiller H. A. Shady J. R. Margittai M. (2023). MAP2 caps tau fibrils and inhibits aggregation. J. Biol. Chem. 299 (7), 104891. 10.1016/j.jbc.2023.104891 37286038 PMC10404690

[B33] Hooper C. Killick R. Lovestone S. (2008). The GSK3 hypothesis of Alzheimer's disease. J. Neurochem. 104 (6), 1433–1439. 10.1111/j.1471-4159.2007.05194.x 18088381 PMC3073119

[B34] Huang M. Bin N. R. Rai J. Ma K. Chow C. H. Eide S. (2023). Neuronal SNAP-23 is critical for synaptic plasticity and spatial memory independently of NMDA receptor regulation. iScience 26 (5), 106664. 10.1016/j.isci.2023.106664 37168570 PMC10165271

[B35] Jedlicka P. Vnencak M. Krueger D. D. Jungenitz T. Brose N. Schwarzacher S. W. (2015). Neuroligin-1 regulates excitatory synaptic transmission, LTP and EPSP-spike coupling in the dentate gyrus *in vivo* . Brain Struct. Funct. 220 (1), 47–58. 10.1007/s00429-013-0636-1 25713840

[B36] Jeong S. (2017). Molecular and cellular basis of neurodegeneration in Alzheimer's disease. Mol. Cells 40 (9), 613–620. 10.14348/molcells.2017.0096 28927263 PMC5638769

[B37] Jeong J. Han W. Hong E. Pandey S. Li Y. Lu W. (2023). Regulation of NLGN3 and the synaptic Rho-GEF signaling pathway by CDK5. J. Neurosci. 43 (44), 7264–7275. 10.1523/JNEUROSCI.2309-22.2023 37699715 PMC10621767

[B38] Jiao S. S. Shen L. L. Zhu C. Bu X. L. Liu Y. H. Liu C. H. (2016). Brain-derived neurotrophic factor protects against tau-related neurodegeneration of Alzheimer's disease. Transl. Psychiatry 6 (10), e907. 10.1038/tp.2016.186 27701410 PMC5315549

[B39] John A. Reddy P. H. (2021). Synaptic basis of Alzheimer's disease: focus on synaptic amyloid beta, P-tau and mitochondria. Ageing Res. Rev. 65, 101208. 10.1016/j.arr.2020.101208 33157321 PMC7770124

[B40] Karimipour M. Rahbarghazi R. Tayefi H. Shimia M. Ghanadian M. Mahmoudi J. (2019). Quercetin promotes learning and memory performance concomitantly with neural stem/progenitor cell proliferation and neurogenesis in the adult rat dentate gyrus. Int. J. Dev. Neurosci. 74, 18–26. 10.1016/j.ijdevneu.2019.02.005 30822517

[B41] Karthikkeyan G. Najar M. A. Pervaje R. Pervaje S. K. Modi P. K. Prasad T. S. K. (2020a). Identification of molecular network associated with neuroprotective effects of yashtimadhu (Glycyrrhiza glabra L.) by quantitative proteomics of rotenone-induced Parkinson's disease model. ACS Omega 5 (41), 26611–26625. 10.1021/acsomega.0c03420 33110989 PMC7581237

[B42] Karthikkeyan G. Pervaje R. Subbannayya Y. Patil A. H. Modi P. K. Prasad T. S. K. (2020b). Plant omics: Metabolomics and network pharmacology of liquorice, Indian ayurvedic medicine yashtimadhu. OMICS 24 (12), 743–755. 10.1089/omi.2020.0156 33275529

[B43] Karthikkeyan G. Pervaje R. Pervaje S. K. Prasad T. S. K. Modi P. K. (2021). Prevention of MEK-ERK-1/2 hyper-activation underlines the neuroprotective effect of Glycyrrhiza glabra L. (yashtimadhu) against rotenone-induced cellular and molecular aberrations. J. Ethnopharmacol. 274, 114025. 10.1016/j.jep.2021.114025 33775804

[B44] Kim J. Jung S. Y. Lee Y. K. Park S. Choi J. S. Lee C. J. (2008). Neuroligin-1 is required for normal expression of LTP and associative fear memory in the amygdala of adult animals. Proc. Natl. Acad. Sci. U. S. A. 105 (26), 9087–9092. 10.1073/pnas.0803448105 18579781 PMC2449369

[B45] Kim S. W. Jin Y. Shin J. H. Kim I. D. Lee H. K. Park S. (2012). Glycyrrhizic acid affords robust neuroprotection in the postischemic brain via anti-inflammatory effect by inhibiting HMGB1 phosphorylation and secretion. Neurobiol. Dis. 46 (1), 147–156. 10.1016/j.nbd.2011.12.056 22266336

[B46] Kitagishi Y. Nakanishi A. Ogura Y. Matsuda S. (2014). Dietary regulation of PI3K/AKT/GSK-3beta pathway in Alzheimer's disease. Alzheimers Res. Ther. 6 (3), 35. 10.1186/alzrt265 25031641 PMC4075129

[B47] Kitamura Y. Shimohama S. Kamoshima W. Ota T. Matsuoka Y. Nomura Y. (1998). Alteration of proteins regulating apoptosis, Bcl-2, Bcl-x, Bax, Bak, Bad, ICH-1 and CPP32, in Alzheimer's disease. Brain Res. 780 (2), 260–269. 10.1016/s0006-8993(97)01202-x 9507158

[B48] Kulkarni R. Girish K. J. Kumar A. (2012). Nootropic herbs (medhya rasayana) in ayurveda: an update. Pharmacogn. Rev. 6 (12), 147–153. 10.4103/0973-7847.99949 23055641 PMC3459457

[B49] Kunii M. Noguchi Y. Yoshimura S. I. Kanda S. Iwano T. Avriyanti E. (2021). SNAP23 deficiency causes severe brain dysplasia through the loss of radial glial cell polarity. J. Cell. Biol. 220 (1), e201910080. 10.1083/jcb.201910080 33332551 PMC7754684

[B50] Lee J. H. Kim K. T. (2004). Induction of cyclin-dependent kinase 5 and its activator p35 through the extracellular-signal-regulated kinase and protein kinase A pathways during retinoic-acid mediated neuronal differentiation in human neuroblastoma SK-N-BE(2)C cells. J. Neurochem. 91 (3), 634–647. 10.1111/j.1471-4159.2004.02770.x 15485494

[B51] Lee C. W. Lau K. F. Miller C. C. Shaw P. C. (2003). Glycogen synthase kinase-3 beta-mediated tau phosphorylation in cultured cell lines. Neuroreport 14 (2), 257–260. 10.1097/00001756-200302100-00020 12598741

[B52] Lee H. G. Casadesus G. Nunomura A. Zhu X. Castellani R. J. Richardson S. L. (2009). The neuronal expression of MYC causes a neurodegenerative phenotype in a novel transgenic mouse. Am. J. Pathol. 174 (3), 891–897. 10.2353/ajpath.2009.080583 19164506 PMC2665749

[B53] Lei H. P. Yang X. Hu Y. T. Wu L. N. Wei A. H. Yu L. (2026). Gastrodin alleviates mitochondrial energy metabolism dysfunction via activating beta-catenin/c-Myc/MCT2 signaling in Alzheimer's disease models. J. Ethnopharmacol. 354, 120548. 10.1016/j.jep.2025.120548 40915373

[B54] Li B. S. Ma W. Jaffe H. Zheng Y. Takahashi S. Zhang L. (2003). Cyclin-dependent kinase-5 is involved in neuregulin-dependent activation of phosphatidylinositol 3-kinase and Akt activity mediating neuronal survival. J. Biol. Chem. 278 (37), 35702–35709. 10.1074/jbc.M302004200 12824184

[B55] Li B. Yamamori H. Tatebayashi Y. Shafit-Zagardo B. Tanimukai H. Chen S. (2008). Failure of neuronal maturation in Alzheimer disease dentate gyrus. J. Neuropathol. Exp. Neurol. 67 (1), 78–84. 10.1097/nen.0b013e318160c5db 18091557 PMC3191920

[B56] Lopez O. L. DeKosky S. T. (2003). Neuropathology of Alzheimer's disease and mild cognitive impairment. Rev. Neurol. 37 (2), 155–163. 12938076

[B57] Luo L. Jin Y. Kim I. D. Lee J. K. (2013). Glycyrrhizin attenuates kainic Acid-induced neuronal cell death in the mouse hippocampus. Exp. Neurobiol. 22 (2), 107–115. 10.5607/en.2013.22.2.107 23833559 PMC3699671

[B58] Marino Gammazza A. Restivo V. Baschi R. Caruso Bavisotto C. Cefalu A. B. Accardi G. (2022). Circulating molecular chaperones in subjects with amnestic mild cognitive impairment and Alzheimer's disease: data from the zabut aging project. J. Alzheimers Dis. 87 (1), 161–172. 10.3233/JAD-180825 30584145 PMC9277667

[B59] Martins N. Barros L. Dueñas M. Santos-Buelga C. Ferreira I. C. (2015). Characterization of phenolic compounds and antioxidant properties of Glycyrrhiza glabra L. rhizomes and roots. RSC Advances 5 (34), 26991–26997. 10.1039/c5ra03963k

[B60] McCommis K. S. Baines C. P. (2012). The role of VDAC in cell death: friend or foe? Biochim. Biophys. Acta 1818 (6), 1444–1450. 10.1016/j.bbamem.2011.10.025 22062421 PMC3288473

[B61] Modi P. K. Komaravelli N. Singh N. Sharma P. (2012). Interplay between MEK-ERK signaling, cyclin D1, and cyclin-dependent kinase 5 regulates cell cycle reentry and apoptosis of neurons. Mol. Biol. Cell. 23 (18), 3722–3730. 10.1091/mbc.E12-02-0125 22833568 PMC3442418

[B62] Nakatani Y. Kobe A. Kuriya M. Hiroki Y. Yahagi T. Sakakibara I. (2017). Neuroprotective effect of liquiritin as an antioxidant via an increase in glucose-6-phosphate dehydrogenase expression on B65 neuroblastoma cells. Eur. J. Pharmacol. 815, 381–390. 10.1016/j.ejphar.2017.09.040 28970010

[B63] Naoi M. Shamoto-Nagai M. Maruyama W. (2019). Neuroprotection of multifunctional phytochemicals as novel therapeutic strategy for neurodegenerative disorders: antiapoptotic and antiamyloidogenic activities by modulation of cellular signal pathways. Future Neurol. 14 (1), FNL9. 10.2217/fnl-2018-0028

[B64] Neill D. Hughes D. Edwardson J. A. Rima B. K. Allsop D. (1994). Human IMR-32 neuroblastoma cells as a model cell line in Alzheimer's disease research. J. Neurosci. Res. 39 (4), 482–493. 10.1002/jnr.490390415 7884825

[B65] Neri-Gomez T. Espinosa-Raya J. Diaz-Cintra S. Segura-Uribe J. Orozco-Suarez S. Gallardo J. M. (2017). Tibolone modulates neuronal plasticity through regulating tau, GSK3beta/Akt/PI3K pathway and CDK5 p35/p25 complexes in the hippocampus of aged male mice. Neural Regen. Res. 12 (4), 588–595. 10.4103/1673-5374.205098 28553339 PMC5436357

[B66] Niikura T. Tajima H. Kita Y. (2006). Neuronal cell death in Alzheimer's disease and a neuroprotective factor, humanin. Curr. Neuropharmacol. 4 (2), 139–147. 10.2174/157015906776359577 18615127 PMC2430668

[B67] Numakawa T. Odaka H. (2021). Brain-derived neurotrophic factor signaling in the pathophysiology of Alzheimer's disease: beneficial effects of flavonoids for neuroprotection. Int. J. Mol. Sci. 22 (11), 5719. 10.3390/ijms22115719 34071978 PMC8199014

[B106] Paradis E. Douillard H. Koutroumanis M. Goodyer C. LeBlanc A. (1996). Amyloid beta peptide of Alzheimer’s disease downregulates Bcl-2 and upregulates bax expression in human neurons. J. Neurosci. 16 (23), 7533–7539. 10.1523/JNEUROSCI.16-23-07533.1996 8922409 PMC6579094

[B68] Pastorino G. Cornara L. Soares S. Rodrigues F. Oliveira M. (2018). Liquorice (glycyrrhiza glabra): a phytochemical and pharmacological review. Phytother. Res. 32 (12), 2323–2339. 10.1002/ptr.6178 30117204 PMC7167772

[B69] Prussing K. Voigt A. Schulz J. B. (2013). *Drosophila melanogaster* as a model organism for Alzheimer's disease. Mol. Neurodegener. 8, 35. 10.1186/1750-1326-8-35 24267573 PMC4222597

[B70] Rajmohan R. Reddy P. H. (2017). Amyloid-beta and phosphorylated tau accumulations cause abnormalities at synapses of Alzheimer's disease neurons. J. Alzheimers Dis. 57 (4), 975–999. 10.3233/JAD-160612 27567878 PMC5793225

[B71] Rao R. R. Kisaalita W. S. (2002). Biochemical and electrophysiological differentiation profile of a human neuroblastoma (IMR-32) cell line. Vitro Cell. Dev. Biol. Anim. 38 (8), 450–456. 10.1290/1071-2690(2002)038<0450:BAEDPO>2.0.CO;2 12605539

[B72] Rojas-Garcia A. Fernandez-Ochoa A. Cadiz-Gurrea M. L. Arraez-Roman D. Segura-Carretero A. (2023). Neuroprotective effects of agri-food By-Products rich in phenolic compounds. Nutrients 15 (2), 449. 10.3390/nu15020449 36678322 PMC9865516

[B73] Ruitenberg M. F. L. (2024). Cognition and movement in neurodegenerative disorders: a dynamic duo. Neural Regen. Res. 19 (10), 2101–2102. 10.4103/1673-5374.392879 38488538 PMC11034590

[B74] Sberna G. Saez-Valero J. Beyreuther K. Masters C. L. Small D. H. (1997). The amyloid beta-protein of Alzheimer's disease increases acetylcholinesterase expression by increasing intracellular calcium in embryonal carcinoma P19 cells. J. Neurochem. 69 (3), 1177–1184. 10.1046/j.1471-4159.1997.69031177.x 9282941

[B75] Schedin-Weiss S. Inoue M. Hromadkova L. Teranishi Y. Yamamoto N. G. Wiehager B. (2017). Monoamine oxidase B is elevated in Alzheimer disease neurons, is associated with gamma-secretase and regulates neuronal amyloid beta-peptide levels. Alzheimers Res. Ther. 9 (1), 57. 10.1186/s13195-017-0279-1 28764767 PMC5540560

[B76] Scheltens P. De Strooper B. Kivipelto M. Holstege H. Chetelat G. Teunissen C. E. (2021). Alzheimer's disease. Lancet 397 (10284), 1577–1590. 10.1016/S0140-6736(20)32205-4 33667416 PMC8354300

[B77] Schmidt F. M. Mergl R. Stach B. Jahn I. Gertz H. J. Schonknecht P. (2014). Elevated levels of cerebrospinal fluid neuron-specific enolase (NSE) in Alzheimer's disease. Neurosci. Lett. 570, 81–85. 10.1016/j.neulet.2014.04.007 24746933

[B78] Schneider C. A. Rasband W. S. Eliceiri K. W. (2012). NIH image to ImageJ: 25 years of image analysis. Nat. Methods 9 (7), 671–675. 10.1038/nmeth.2089 22930834 PMC5554542

[B79] Serrano-Pozo A. Frosch M. P. Masliah E. Hyman B. T. (2011). Neuropathological alterations in Alzheimer disease. Cold Spring Harb. Perspect. Med. 1 (1), a006189. 10.1101/cshperspect.a006189 22229116 PMC3234452

[B80] Shao S. Lu H. Zu R. Chen Y. Peng Z. Peng Q. (2026). Pharmacological regulation of mitophagy by natural plant products as a therapeutic target for Alzheimer's disease. Phytother. Res. 40 (1), 248–266. 10.1002/ptr.70131 41272400

[B81] Sharma P. Pathak P. Tyagi V. Khan F. Shanker K. Darokar M. P. (2022). Investigation of the potential of Glycyrrhiza glabra as a bioavailability enhancer of vitamin B12. Front. Nutr. 9, 1038902. 10.3389/fnut.2022.1038902 36386946 PMC9650095

[B82] Sheshagiri S. Patel K. S. Rajagopala S. (2015). Randomized placebo-controlled clinical study on enhancement of Medha (intelligence quotient) in school going children with Yahstimadhu granules. Ayu 36 (1), 56–62. 10.4103/0974-8520.169011 26730140 PMC4687241

[B83] Singh S. K. Gaur R. Kumar A. Fatima R. Mishra L. Srikrishna S. (2014). The flavonoid derivative 2-(4' Benzyloxyphenyl)-3-hydroxy-chromen-4-one protects against Abeta42-induced neurodegeneration in transgenic Drosophila: insights from *in silico* and *in vivo* studies. Neurotox. Res. 26 (4), 331–350. 10.1007/s12640-014-9466-z 24706035

[B84] Soderberg L. Johannesson M. Nygren P. Laudon H. Eriksson F. Osswald G. (2023). Lecanemab, aducanumab, and gantenerumab - binding profiles to different forms of amyloid-beta might explain efficacy and side effects in clinical trials for alzheimer's disease. Neurotherapeutics 20 (1), 195–206. 10.1007/s13311-022-01308-6 36253511 PMC10119362

[B85] Soni U. Singh K. Jain D. Pujari R. (2025). Exploring Alzheimer's disease treatment: established therapies and novel strategies for future care. Eur. J. Pharmacol. 998, 177520. 10.1016/j.ejphar.2025.177520 40097131

[B86] Su J. H. Zhao M. Anderson A. J. Srinivasan A. Cotman C. W. (2001). Activated caspase-3 expression in Alzheimer's and aged control brain: correlation with Alzheimer pathology. Brain Res. 898 (2), 350–357. 10.1016/s0006-8993(01)02018-2 11306022

[B87] Sun Y. C. (2009). Examination of effects of GSK3beta phosphorylation, beta-catenin phosphorylation, and beta-catenin degradation on kinetics of wnt signaling pathway using computational method. Theor. Biol. Med. Model. 6, 13. 10.1186/1742-4682-6-13 19622173 PMC2724396

[B88] Tarsa L. Goda Y. (2002). Synaptophysin regulates activity-dependent synapse formation in cultured hippocampal neurons. Proc. Natl. Acad. Sci. U. S. A. 99 (2), 1012–1016. 10.1073/pnas.022575999 11792847 PMC117422

[B89] Teixeira A. L. Rocha N. P. Gatchel J. (2023). Behavioral or neuropsychiatric symptoms of Alzheimer's disease: from psychopathology to pharmacological management. Arq. Neuro-Psiquiatria 81 (12), 1152–1162. 10.1055/s-0043-1777774 38157881 PMC10756775

[B90] Teng L. Meng Q. Lu J. Xie J. Wang Z. Liu Y. (2014). Liquiritin modulates ERK- and AKT/GSK-3beta-dependent pathways to protect against glutamate-induced cell damage in differentiated PC12 cells. Mol. Med. Rep. 10 (2), 818–824. 10.3892/mmr.2014.2289 24888902 PMC4094771

[B91] Tolar M. Abushakra S. Hey J. A. Porsteinsson A. Sabbagh M. (2020). Aducanumab, gantenerumab, BAN2401, and ALZ-801-the first wave of amyloid-targeting drugs for Alzheimer's disease with potential for near term approval. Alzheimers Res. Ther. 12 (1), 95. 10.1186/s13195-020-00663-w 32787971 PMC7424995

[B92] Ullah A. Munir S. Badshah S. L. Khan N. Ghani L. Poulson B. G. (2020). Important flavonoids and their role as a therapeutic agent. Molecules 25 (22), 5243. 10.3390/molecules25225243 33187049 PMC7697716

[B93] Victor Atoki A. Aja P. M. Shinkafi T. S. Ondari E. N. Adeniyi A. I. Fasogbon I. V. (2025). Exploring the versatility of *Drosophila melanogaster* as a model organism in biomedical research: a comprehensive review. Fly. (Austin) 19 (1), 2420453. 10.1080/19336934.2024.2420453 39722550 PMC11702942

[B94] Vitek G. E. Decourt B. Sabbagh M. N. (2023). Lecanemab (BAN2401): an anti-beta-amyloid monoclonal antibody for the treatment of Alzheimer disease. Expert Opin. Investig. Drugs 32 (2), 89–94. 10.1080/13543784.2023.2178414 36749830 PMC10275297

[B95] Wahab S. Annadurai S. Abullais S. S. Das G. Ahmad W. Ahmad M. F. (2021). Glycyrrhiza glabra (licorice): a comprehensive review on its phytochemistry, biological activities, clinical evidence and toxicology. Plants (Basel) 10 (12), 2751. 10.3390/plants10122751 34961221 PMC8703329

[B96] Walls K. C. Coskun P. Gallegos-Perez J. L. Zadourian N. Freude K. Rasool S. (2012). Swedish Alzheimer mutation induces mitochondrial dysfunction mediated by HSP60 mislocalization of amyloid precursor protein (APP) and beta-amyloid. J. Biol. Chem. 287 (36), 30317–30327. 10.1074/jbc.M112.365890 22753410 PMC3436283

[B97] Wang L. Yang R. Yuan B. Liu Y. Liu C. (2015). The antiviral and antimicrobial activities of licorice, a widely-used Chinese herb. Acta Pharm. Sin. B 5 (4), 310–315. 10.1016/j.apsb.2015.05.005 26579460 PMC4629407

[B98] Wu X. Yang Z. Zou J. Gao H. Shao Z. Li C. (2025). Protein kinases in neurodegenerative diseases: current understandings and implications for drug discovery. Signal Transduct. Target Ther. 10 (1), 146. 10.1038/s41392-025-02179-x 40328798 PMC12056177

[B99] Yang E. J. Park G. H. Song K. S. (2013). Neuroprotective effects of liquiritigenin isolated from licorice roots on glutamate-induced apoptosis in hippocampal neuronal cells. Neurotoxicology 39, 114–123. 10.1016/j.neuro.2013.08.012 24012889

[B100] Yang Z. Wang Y. Zhang Y. He X. Zhong C. Q. Ni H. (2018). RIP3 targets pyruvate dehydrogenase complex to increase aerobic respiration in TNF-induced necroptosis. Nat. Cell. Biol. 20 (2), 186–197. 10.1038/s41556-017-0022-y 29358703

[B101] Yang Z. Zou Y. Wang L. (2023). Neurotransmitters in prevention and treatment of alzheimer's disease. Int. J. Mol. Sci. 24 (4), 3841. 10.3390/ijms24043841 36835251 PMC9966535

[B102] Yang J. Zhao H. Qu S. (2024). Phytochemicals targeting mitophagy: therapeutic opportunities and prospects for treating Alzheimer's disease. Biomed. Pharmacother. 177, 117144. 10.1016/j.biopha.2024.117144 39004063

[B103] Youle R. J. Strasser A. (2008). The BCL-2 protein family: opposing activities that mediate cell death. Nat. Rev. Mol. Cell. Biol. 9 (1), 47–59. 10.1038/nrm2308 18097445

[B104] Yu X. Y. Lin S. G. Zhou Z. W. Chen X. Liang J. Yu X. Q. (2007). Role of P-glycoprotein in limiting the brain penetration of glabridin, an active isoflavan from the root of Glycyrrhiza glabra. Pharm. Res. 24 (9), 1668–1690. 10.1007/s11095-007-9297-1 17551811

[B105] Zhang H. Han Y. Zhang L. Jia X. Niu Q. (2021). The GSK-3beta/beta-Catenin signaling-mediated brain-derived neurotrophic factor pathway is involved in aluminum-induced impairment of hippocampal LTP *in vivo* . Biol. Trace Elem. Res. 199 (12), 4635–4645. 10.1007/s12011-021-02582-9 33462795

